# The quantum-optics Hamiltonian in the Multipolar gauge

**DOI:** 10.1038/s41598-017-11076-5

**Published:** 2017-09-11

**Authors:** Emmanuel Rousseau, Didier Felbacq

**Affiliations:** Université de Montpellier, Laboratoire Charles Coulomb UMR 5221, F-34095 Montpellier, France

## Abstract

This article deals with the fundamental problem of light-matter interaction in the quantum theory. Although it is described through the vector potential in quantum electrodynamics, it is believed by some that a hamiltonian involving only the electric and the magnetic fields is preferable. In the literature this hamiltonian is known as the Power-Zienau-Woolley hamiltonian. We question its validity and show that it is not equivalent to the minimal-coupling hamiltonian. In this article, we show that these two hamiltonians are not connected through a gauge transformation. We find that the gauge is not fixed in the Power-Zienau-Woolley hamiltonian. The interaction term is written in one gauge whereas the rest of the hamiltonian is written in another gauge. The Power-Zienau-Woolley hamiltonian and the minimal-coupling one are related through a unitary transformation that does not fulfill the gauge fixing constraints. Consequently, they predict different physical results. In this letter, we provide the correct quantum theory in the multipolar gauge with a hamiltonian involving only the physical fields.

## Introduction

The essence of quantum optics is to describe the interaction between light and matter, both treated at the quantum level. The starting point of the quantum optics theory is the minimal-coupling hamiltonian^1^ that describes the coupling between matter and the electromagnetic field through the potentials, $\overrightarrow{A}(\overrightarrow{x},t)$ and $\varphi (\overrightarrow{x},t)$, rather than through the electric field $\overrightarrow{E}(\overrightarrow{x},t)$ and the magnetic field $\overrightarrow{B}(\overrightarrow{x},t)$. The electric and the magnetic fields are physically relevant on the contrary to the potentials that depend on a gauge condition. Thus, it has been recognized as useful to find a quantum hamiltonian describing light-matter interactions only through the electric and the magnetic fields. Such a hamiltonian, named the Power-Zienau-Woolley hamiltonian, has been exhibited^2–4^ long ago. It is recognized to be equivalent to the minimal-coupling hamiltonian since both hamiltonians are related through a gauge transformation^4–7^ or through a unitary transformation^1, 2, 4, 8–11^. These two starting points should give exactly the same results but the Power-Zienau-Woolley hamiltonian might be more convenient since it involves only the physical fields. As a consequence, the Power-Zienau-Woolley hamiltonian is widely used in many fields of physics where light and matter interact at the quantum level. To quote only a few of them, one can cite the propagation of light in cold-atoms gas^12^, the quantization of light in dielectric media^13^, the interaction of light with electron gas in semiconducting heterostructures^14^, the description of metamaterials properties^15, 16^, the interaction of light with bulk plasmon^17^, the interaction of light in the near-field^18^, the description of non-linear optical properties^19^ and also the phenomenon of resonance energy transfer at the molecular level^5, 20, 21^. The latter can find applications in biological systems such as chromophores subject to photosynthesis^22^. Recently a letter^23^ extended the validity of the Power-Zienau-Woolley hamiltonian to the case of cavity quantum-electrodynamics. These authors pointed out that the Power-Zienau-Woolley hamiltonian eliminates the *A* ^2^ problem that appears in the minimal-coupling hamiltonian since that term does not appear explicitly in the Power-Zienau-Woolley hamiltonian. As a result, simple hamiltonian models for light-matter interaction in cavities, such as the Jaynes-Cumming hamiltonian^24^ or the Dicke hamiltonian, are valid for a large range of physical parameters^23^. This non-exhaustive list demonstrates that the Power-Zienau-Woolley hamiltonian written in term of physical fields is very useful in many domain of physics. As a consequence it is reproduced in many textbooks of quantum optics^1, 10, 11, 13, 18, 25^.

In this article, we revisit the gauge transformation that produces the Power-Zienau-Woolley hamiltonian from the minimal-coupling hamiltonian^1, 4–7^. We fix the gauge to the Poincaré gauge^26^ (also named the multipolar gauge). The multipolar gauge has the important property that the scalar potential and the vector potential can be written with the help of the physical fields. As a consequence, the hamiltonian we have derived involves only the electric field and the magnetic field. We compute the commutation rules in the Poincaré gauge. This fundamental question is evaded in many references and our main conclusion is that the commutators in the multipolar gauge differ from the commutation rules in the Coulomb gauge. We demonstrate that if the gauge transformation is performed correctly it doesn’t lead to the Power-Zienau-Woolley hamiltonian on the opposite to previous authors conclusions^4–7^ and [ref. 1, p. 333]. Our first conclusion is that the Power-Zienau-Woolley is not related to the minimal-coupling hamiltonian through a gauge transformation. Next, we question the derivation of the Power-Zienau-Woolley hamiltonian from a unitary transformation applied to the minimal-coupling hamiltonian written in the Coulomb gauge (this is the historical derivation of the Power-Zienau-Woolley hamiltonian^2^). We show that cares should be taken when applying a unitary transformation on the minimal-coupling hamiltonian because quantum-electrodynamics is a constrained theory (constrained at least by the gauge conditions). We show that the unitary transformation used by Power et coworkers^2, 4–7, 9, 27, 28^ (see also [ref. 1, p. 282]) modifies the vector potential and its gauge constraint. As a consequence, the interaction term in the Power-Zienau-Woolley hamiltonian is written in the Poincaré gauge whereas the electromagnetic-energy term is written in the Coulomb gauge. It results the generation of non-physical photon states with longitudinal polarization. As a consequence, we conclude that, because of the constraints, the Power-Zienau-Woolley hamiltonian is not unitarily equivalent to the minimal-coupling hamiltonian since these two hamiltonians predict different physical results.

## Results

### The quantum hamiltonian in the multipolar gauge

We start this paper by deriving the quantum hamiltonian and the commutation rules in the multipolar gauge. We start the derivation from a semiclassical lagrangian density. The lagrangian density is a gauge invariant quantity. A local phase-transformation of the Schrödinger field has to be compensated by a transformation of the potential fields in order to leave invariant the equations of motion (see *e*.*g*. [ref. 29, p. 44]). The gauge transformation reads^26^:

1 $${\psi }_{p}(\overrightarrow{x},t)={e}^{i\frac{q}{\hslash }\chi (\overrightarrow{x},t)}\,{\psi }_{c}(\overrightarrow{x},t)$$

2 $${\overrightarrow{A}}_{p}(\overrightarrow{x},t)={\overrightarrow{A}}_{c}(\overrightarrow{x},t)+\overrightarrow{\nabla }[\chi (\overrightarrow{x},t)]$$

3 $${\varphi }_{p}(\overrightarrow{x},t)={\varphi }_{c}(\overrightarrow{x},t)-\frac{\partial }{\partial t}\chi (\overrightarrow{x},t),$$

where *q* is the electron charge, ${\overrightarrow{A}}_{p}(\overrightarrow{x},t)$ is the vector potential in the final gauge “*p*” (Poincaré gauge here) and ${\overrightarrow{A}}_{c}(\overrightarrow{x},t)$ is the vector potential in the initial gauge “*c*” (Coulomb gauge here). The scalar potential is $\varphi (\overrightarrow{x},t)$ and $\chi (\overrightarrow{x},{\overrightarrow{A}}_{c},t)$ is the gauge function that transforms one gauge into another. Gauges are characterized by gauge conditions that constrain the possible values of the potentials^30^. Well-known gauge conditions are the Coulomb gauge condition $\overrightarrow{\nabla }\mathrm{.}{\overrightarrow{A}}_{c}=0$ or the Lorentz gauge condition $\overrightarrow{\nabla }\mathrm{.}{\overrightarrow{A}}_{l}+\frac{1}{c}\frac{\partial {\varphi }_{l}}{\partial t}=0$. In this work, we use the Poincaré-gauge condition that reads $\overrightarrow{x}\mathrm{.}{\overrightarrow{A}}_{p}(\overrightarrow{x},t)=0$. As a consequence of this gauge condition, the scalar potential and the vector potential can be explicitly written in term of the physical fields^26^:

4 $${\varphi }_{p}(\overrightarrow{x},t)=-\overrightarrow{x}\mathrm{.}{\int }_{0}^{1}\,\overrightarrow{E}(u\overrightarrow{x},t)du$$

5 $${\overrightarrow{A}}_{p}(\overrightarrow{x},t)=-\overrightarrow{x}\times {\int }_{0}^{1}\,\overrightarrow{B}(u\overrightarrow{x},t)udu$$

The lagrangian density that describes a non-relativistic charged particle without spin and with electric charge *q*, evolving in a potential $V(\overrightarrow{x})$, for example created by the nucleus of an atom, reads^31^:

6 $$\begin{array}{rcl} {\mathcal L} (\overrightarrow{x},t) & = & i\hslash {\psi }^{* }(\overrightarrow{x},t)\,[{\partial }_{t}+\frac{iq}{\hslash }\varphi (\overrightarrow{x},t)]\,\psi (\overrightarrow{x},t)\\  &  & -\frac{{\hslash }^{2}}{2m}\,[{\partial }_{\mu }+\frac{iq}{\hslash }{A}_{\mu }(\overrightarrow{x},t)]\,{\psi }^{* }(\overrightarrow{x},t)\,[{\partial }^{\mu }-\frac{iq}{\hslash }{A}^{\mu }(\overrightarrow{x},t)]\,\psi (\overrightarrow{x},t)\\  &  & -{\psi }^{* }\,(\overrightarrow{x},t)\,V(\overrightarrow{x})\,\psi (\overrightarrow{x},t)\\  &  & +\frac{1}{2}{\varepsilon }_{0}\,{[{\partial }_{t}\overrightarrow{A}(\overrightarrow{x},t)+\overrightarrow{\nabla }\varphi (\overrightarrow{x},t)]}^{2}-\frac{1}{2{\mu }_{0}}{\overrightarrow{B}}^{2}(\overrightarrow{x},t)\end{array}$$

The Einstein summation convention is used for the space variables *μ* = 1, 2, 3, but we discriminate the time variable since we work in a non-relativistic approximation, which is valid for quantum optics.

From the lagrangian density, we can compute the canonical conjugate momenta associated to each dynamical variables that are $\psi (\overrightarrow{x},t)$, ${A}_{\mu }(\overrightarrow{x},t)$ and $\varphi (\overrightarrow{x},t)$. The canonical momentum associated to *ψ*(*x*, *t*) is given by ${\pi }_{\psi }=\frac{\partial  {\mathcal L} }{\partial {\partial }_{t}\psi }=i\hslash {\psi }^{* }(x,t)$ whereas the canonical momentum associated to *A* ^*μ*^ is:

7 $${\pi }_{\mu }(\overrightarrow{x},t)=\frac{\partial  {\mathcal L} }{\partial {\partial }_{t}{A}^{\mu }}={\varepsilon }_{0}({\partial }_{t}{A}_{\mu }+{\partial }_{\mu }\varphi )=-{\varepsilon }_{0}{E}_{\mu }(\overrightarrow{x},t)$$

The canonical momentum conjugated to the vector potential $\overrightarrow{A}$ is the electric field (up to the constant −*ε*_0_). This result is then gauge invariant because of the gauge invariance of the electric field. The canonical momentum associated to the scalar potential is null *π*_*ϕ*_ = 0. With the help of the canonical momenta, the hamiltonian density can be derived. After an integration by part it reads (see [ref. 32, p. 74]):

8 $$\begin{array}{rcl} {\mathcal H} (\overrightarrow{x},t) & = & \frac{1}{i\hslash }{\pi }_{\psi }(\overrightarrow{x},t)\,[(-\frac{{\hslash }^{2}}{2m})\,{({\partial }_{\mu }-\frac{iq}{\hslash }{A}_{\mu })}^{2}+V(\overrightarrow{x},t)]\,\psi (\overrightarrow{x},t)\\  &  & +[\frac{1}{2{\varepsilon }_{0}}{\pi }_{\mu }(\overrightarrow{x},t){\pi }^{\mu }(\overrightarrow{x},t)+\frac{1}{2{\mu }_{0}}{B}_{\mu }(\overrightarrow{x},t)\,{B}^{\mu }(\overrightarrow{x},t)]\\  &  & +[{\partial }_{\mu }{\pi }^{\mu }(\overrightarrow{x},t)+\frac{q}{i\hslash }{\pi }_{\psi }(\overrightarrow{x},t)\,\psi (\overrightarrow{x},t)]\,\varphi (\overrightarrow{x},t)\end{array}$$

with the help of this Hamiltonian density, Hamilton equations give the dynamical equations^31^, p.374, *i*.*e*. the time-evolution of the vector potential [that is in fact the eq. (7)], the Maxwell-Ampère equation and the Schrödinger equation. However, in the hamiltonian formalism, at the semiclassical level as well as in the quantum theory, the Maxwell-Gauss equation is considered as a constraint that the solutions have to satisfy (see [ref. 31, p. 363, 375]).

**Figure 1:**
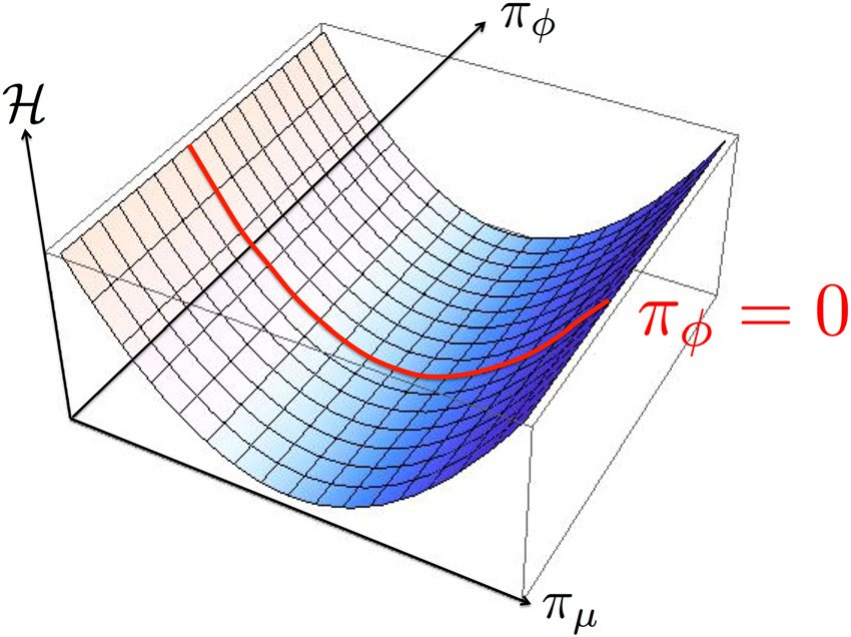
Schematic view of the action of the Dirac brackets. The hamiltonian density varies as ${\mathcal H} \propto {\pi }_{\mu }^{2}$. We plot its restriction to the phase space {*π*_*μ*_, *π*_*ϕ*_}. Because of the constraint *π*_*ϕ*_ = 0, the accessible values of the hamiltonian density are given by the red line. The Dirac brackets restrict the dynamics to the red line whereas the hamiltonian density is defined in the entire phase-space {*π*_*μ*_, *π*_*ϕ*_}.

We are now ready to quantize the theory. We quantize both the Schrödinger field and the electromagnetic field. Following the canonical quantization procedure^33–35^, we can obtain a quantum theory by imposing canonical commutation relations between canonical conjugated quantities now considered as operators acting on a Hilbert space. In the Heisenberg picture, the time-evolution of operators is given by the Heisenberg equation. The generator of time-translations is the hamiltonian operator defined on the phase-space spanned by the dynamical variables $\{(\psi ,{\pi }_{\psi });{({A}^{\mu },{\pi }_{\mu })}_{\mu =1,2,3};(\varphi ,{\pi }_{\phi })\}$. All these quantities are considered as being independent of each others. This is indeed not correct for quantum electrodynamics that is a constrained theory. The original idea of Dirac^33^ is to restrict the phase-space to the subspace allowed by the constraints with the help of the commutators. Then, the commutators allow to derive the “correct” equations between operators; “correct” means here that, as a postulate, the quantum operators have to satisfy the same analytical relationships as the classical quantities and the same constraints^33, 35^. To this goal, Dirac^33^ defines new brackets^33, 35^, the Dirac brackets noted {●, ○}_*D*_, that replace the Poisson brackets {●, ○}_*P*_. The commutators are obtained by multiplying the Dirac brackets by *iħ*, $[\hat{\bullet },\hat{\circ }]=i\hslash {\{\bullet ,\circ \}}_{D}\hat{I}$ where $\hat{I}$ is the identity matrix. The Dirac brackets force the dynamical equations to follow the constraints as illustrated schematically in Fig. 1. There, the Hamiltonian density eq. (8) is plotted as a function of *π*_*μ*_ and *π*_*ϕ*_. With the help of the Heisenberg equation, the dynamical equation for the operator ${\hat{\pi }}_{\varphi }(\overrightarrow{x},t)$ is: $i\hslash {\dot{\hat{\pi }}}_{\varphi }(\overrightarrow{x},t)$ = $i\hslash \int \,d\overrightarrow{y}({\partial }_{\mu }{\hat{\pi }}^{\mu }(\overrightarrow{y},t)+\tfrac{q}{i\hslash }{\hat{\pi }}_{\psi }(\overrightarrow{y},t)\,\hat{\psi }(\overrightarrow{y},t))$ ${\{{\pi }_{\varphi }(\overrightarrow{x},t),\varphi (\overrightarrow{y},t)\}}_{D}\hat{I}$. It would not be zero for independent variables. Indeed, for a couple of independent dynamical variables *α* and its canonical momentum *π*_*α*_, the Dirac bracket value is given by the Poisson bracket value ${\{\alpha (\overrightarrow{x},t),{\pi }_{\alpha }(\overrightarrow{y},t)\}}_{D}$ = ${\{\alpha (\overrightarrow{x},t),{\pi }_{\alpha }(\overrightarrow{y},t)\}}_{P}$ = $\delta (\overrightarrow{x}-\overrightarrow{y})$. In the present theory, the constraint is ensured by the Dirac bracket ${\{{\hat{\pi }}_{\varphi }(\overrightarrow{x},t),\varphi (\overrightarrow{y},t)\}}_{D}=0$ that projects the dynamical equation on the red line in Fig. 1 given by ${\hat{\pi }}_{\varphi }=0$.

Dirac brackets are fundamental quantities to build up a constrained quantum theory. We now review the constraints that act on the quantum optics theory. Four constraints denoted ${\chi }_{i}(\overrightarrow{x},t)$ (*i* = 1 … 4) act on the dynamics of the system. The canonical conjugate momentum of the scalar field being null, the first constraint is ${\chi }_{1}(\overrightarrow{x},t)={\pi }_{\varphi }(\overrightarrow{x},t)$. As this constraint has to hold at any time, a second constraint derives: ${\chi }_{2}(\overrightarrow{x},t)=\overrightarrow{\nabla }\,\mathrm{.}\,\pi (\overrightarrow{x},t)+\frac{q}{i\hslash }{\pi }_{\psi }(\overrightarrow{x},t)\,\psi (\overrightarrow{x},t)$, this is in fact Maxwell-Gauss equation. The third constraint is the gauge condition that we chose to be the Poincaré-gauge condition ${\chi }_{3}(\overrightarrow{x},t)=\overrightarrow{x}\,{\rm{and}}\,\overrightarrow{A}(\overrightarrow{x},t)$. The last constraint is ${\chi }_{4}(\overrightarrow{x},t)=\overrightarrow{x}\mathrm{.[}\overrightarrow{\pi }(\overrightarrow{x},t)/{\varepsilon }_{0}-\overrightarrow{\nabla }\varphi \,(\overrightarrow{x},t)]$ deriving from ${\chi }_{3}(\overrightarrow{x},t)$ since it has to hold at all time. These four constraints allow to compute the Dirac brackets that give the canonical commutation relations between quantum operators. We impose canonical anti-commutation relations for the Schrödinger field $\hat{\psi }$ and commutation relations for the electromagnetic field:

9 $$\begin{array}{rcl}\,{[\hat{\psi }(\overrightarrow{x},t),{\hat{\pi }}_{\psi }(\overrightarrow{y},t)]}_{+} & = & i\hslash \delta (\overrightarrow{x}-\overrightarrow{y})\\ \,[{\hat{A}}^{\mu }(\overrightarrow{x},t),{\hat{\pi }}_{\nu }(\overrightarrow{y},t)] & = & i\hslash \,[{\delta }_{\nu }^{\mu }\delta (\overrightarrow{x}-\overrightarrow{y})+{y}_{\nu }{\partial }_{\overrightarrow{x}}^{\mu }{K}_{\mathrm{2,3}}(\overrightarrow{x},\overrightarrow{y})]\\ \,[\hat{\psi }(\overrightarrow{x},t),{\hat{\pi }}^{\mu }(\overrightarrow{y},t)] & = & -q{y}^{\mu }{K}_{\mathrm{2,3}}(\overrightarrow{x},\overrightarrow{y})\hat{\psi }(\overrightarrow{x},t)\\ \,[{\hat{\pi }}_{\psi }(\overrightarrow{x},t),{\hat{\pi }}^{\mu }(\overrightarrow{y},t)] & = & q{y}^{\mu }{K}_{\mathrm{2,3}}(\overrightarrow{x},\overrightarrow{y}){\hat{\pi }}_{\psi }(\overrightarrow{x},t)\end{array}$$

There, the Schwartz distribution ${K}_{\mathrm{2,3}}(\overrightarrow{x},\overrightarrow{y})=\frac{1}{|\overrightarrow{x}|}\theta (|\overrightarrow{x}|-|\overrightarrow{y}|)\,\delta ({x}_{2}-{y}_{2})\,\delta ({x}_{3}-{y}_{3})$ is the solution of the equation ${\overrightarrow{\nabla }}_{\overrightarrow{x}}\mathrm{.[}\overrightarrow{x}{K}_{\mathrm{2,3}}(\overrightarrow{x},\overrightarrow{y})]=\delta (\overrightarrow{x}-\overrightarrow{y})$ and *θ* denotes the Heaviside function. Note that the transverse Dirac distribution that appears in the case of the Coulomb gauge^1, 35^ is not present here. This is the consequence of a different gauge condition, the gauge constraint being taken into account by the commutators. To the best of our knowledge, the commutators in the Poincaré gauge [eq. (9)] are not available in the literature. We provide a detailed calculation of these commutators in the Supplementary Materials (III) where we have also shown that they allow to recover the correct dynamical equations while ensuring the gauge condition $\overrightarrow{x}\,\,\mathrm{.}\,\hat{A}(\overrightarrow{x},t)=0$ [see section (III-F) in the Supplementary Materials].

We can now explicitly write the Hamiltonian-density operator taking into account all the constraints of the Poincaré. With the help of the set of equations [eqs (4), (5) and (7)] and the constraints ${\chi }_{i}(\overrightarrow{x},t)=0$. It reads:

10 $$\begin{array}{rcl}\hat{ {\mathcal H} }(\overrightarrow{x},t) & = & {\hat{\psi }}^{* }(\overrightarrow{x},t)\,\{(-\frac{{\hslash }^{2}}{2m})\,{[\overrightarrow{\nabla }+\frac{iq}{\hslash }\overrightarrow{x}\times {\int }_{0}^{1}\hat{B}(u\overrightarrow{x})udu]}^{2}\}\,\hat{\psi }(\overrightarrow{x},t)\\  &  & +{\hat{\psi }}^{* }(\overrightarrow{x},t)\,V(\overrightarrow{x},t)\,\hat{\psi }(\overrightarrow{x},t)\\  &  & +[\frac{1}{2}{\varepsilon }_{0}{\hat{E}}^{2}(\overrightarrow{x},t)+\frac{1}{2{\mu }_{0}}{\hat{B}}^{2}(\overrightarrow{x},t)]\end{array}$$

The quantum theory in the Poincaré gauge is based on the knowledge of the quantum hamiltonian eq. (10) and the commutation rules eq. (9) that specifies the operators algebra. The hamiltonian involves only the physical fields that can make it suitable for many studies in quantum optics.

### The Power-Zienau-Woolley hamiltonian as a result of a gauge transformation of the minimal-coupling hamiltonian

The Power-Zienau-Woolley hamiltonian is said to be the minimal-coupling hamiltonian in the multipolar gauge^4–7^ (see also [ref. 1, p. 333]). As a consequence our result should be the Power-Zienau-Woolley hamiltonian. But, it is not. For completeness, we quote here Power-Zienau-Woolley results for a hamiltonian density^8^ (see also [ref. 11, p. 97, eq. (4.66)]). In the work of Power *et al*., the canonical momentum associated to the vector potential is found to be the opposite of the transverse part of the displacement vector^5, 7, 8^:

11 $${\overrightarrow{\pi }}_{{\rm{pzw}}}(\overrightarrow{x},t)=-{\overrightarrow{D}}^{\perp }(\overrightarrow{x},t)$$

where ${\overrightarrow{D}}^{\perp }(\overrightarrow{x},t)={\varepsilon }_{0}{\overrightarrow{E}}^{\perp }(\overrightarrow{x},t)+{\overrightarrow{P}}^{\perp }(\overrightarrow{x},t)$ is the transverse part of the displacement vector defined as the sum of the transverse part of the electric field ${\hat{E}}^{\perp }(\overrightarrow{x},t)$ and the transverse part of the polarization field ${\overrightarrow{P}}^{\perp }(\overrightarrow{x},t)$. In our derivation, we have found that the canonical momentum conjugated to the potential vector eq. (7) is proportional to the electric field, which is a gauge-invariant quantity.

The Power-Zienau-Woolley semiclassical-Hamiltonian density reads;

12 $$\begin{array}{rcl}{ {\mathcal H} }_{{\rm{pzw}}}(\overrightarrow{x},t) & = & {\xi }^{* }(\overrightarrow{x},t)\,\{(-\frac{{\hslash }^{2}}{2m})\,{[\overrightarrow{\nabla }+\frac{iq}{\hslash }\overrightarrow{x}\times {\int }_{0}^{1}\overrightarrow{B}(u\overrightarrow{x})udu]}^{2}\}\,\xi (\overrightarrow{x},t)\end{array}$$

13 $$\begin{array}{lll} &  & +{\xi }^{* }(\overrightarrow{x},t)\,V(\overrightarrow{x},t)\,\xi (\overrightarrow{x},t)\end{array}$$

14 $$\begin{array}{lll} &  & +[\frac{1}{2{\varepsilon }_{0}}{\overrightarrow{\pi }}_{{\rm{pzw}}}^{2}(\overrightarrow{x},t)+\frac{1}{2{\mu }_{0}}{\overrightarrow{B}}^{2}(\overrightarrow{x},t)]\end{array}$$

15 $$\begin{array}{lll} &  & +{\xi }^{* }(\overrightarrow{x},t)\{\int \,d\overrightarrow{x}^{\prime} \frac{{\xi }^{* }(\overrightarrow{x}^{\prime} ,t)\,\xi (\overrightarrow{x}^{\prime} ,t)}{4\pi {\varepsilon }_{0}|\overrightarrow{x}-\overrightarrow{x}^{\prime} |}\}\,\xi (\overrightarrow{x},t)\end{array}$$

16 $$\begin{array}{lll} &  & +\frac{1}{{\varepsilon }_{0}}{\overrightarrow{\pi }}_{{\rm{pzw}}}(\overrightarrow{x},t)\,\mathrm{.}\,{\overrightarrow{P}}^{\perp }(\overrightarrow{x},t)\end{array}.$$

17 $$\begin{array}{lll} &  & +\frac{1}{2{\varepsilon }_{0}}{\overrightarrow{P}}^{\perp }{(\overrightarrow{x},t)}^{2}\end{array}$$

where $\xi (\overrightarrow{x},t)$ is the particle wave-function. The kinetic energy term eq. (12) and the potential energy terms eq. (13) in the Power-Zienau-Woolley Hamiltonian density are similar to our result [compare with the two first-terms in eq. (10)]. Nevertheless, the Power-Zienau-Woolley hamiltonian density presents many differences as compared to our result. One of them is the term eq. (14) = $\frac{1}{2{\varepsilon }_{0}}{\overrightarrow{D}}^{2}(\overrightarrow{x},t)+\frac{1}{2{\mu }_{0}}{\overrightarrow{B}}^{2}(\overrightarrow{x},t)$ that is interpreted as the electromagnetic field energy-density. This is awkward since this term is neither the electromagnetic energy in vacuum nor in matter^30^. As far as we know, this point has never been commented. Another difference with our result is that the Power-Zienau-Woolley Hamiltonian density presents an interaction term eq. (16) that couples the displacement vector and the matter through the polarization field. Finally, on the opposite to our results the Power-Zienau-Woolley Hamiltonian density is also characterized by a contact term eq. (17) and a self-interaction term eq. (15). After quantization of the Power-Zienau-Woolley hamiltonian density, the canonical commutations rules are unchanged as compared to the Coulomb gauge^7, 8^. They involve the transverse Dirac-distribution^1, 35^. We have shown that the commutators take the gauge conditions into account. Commutators involving the transverse-Dirac distribution force the dynamics to follow the Coulomb-gauge condition and not the Poincaré-gauge condition. So, in addition to the breaking of the gauge-invariance of the canonical momentum conjugated to the vector potential, we conclude that the Power-Zienau-Woolley hamiltonian density is not the minimal-coupling hamiltonian in the multipolar gauge. In this article, we have focus the discussion on a gauge transformation of the hamiltonian density. In the literature the gauge transformation was done on the hamiltonian function^4–7^. We discuss the gauge transformation for a hamiltonian function in the supplementary materials (I). Again we have found that the Power-Zienau-Woolley hamiltonian is not the minimal-coupling hamiltonian in the Poincaré gauge on the opposite to previous authors conclusions^1, 4–7^. We have also listed in the section (I-C) of the supplementary materials the errors done by previous authors^7^ that leads to the incorrect result.

### The Power-Zienau-Woolley hamiltonian as a result of a unitary transformation of the minimal-coupling hamiltonian

The historical derivation of the Power-Zienau-Woolley hamiltonian^2^ is based on a unitary transformation applied to the minimal-coupling hamiltonian written in the Coulomb gauge. Applying a unitary transformation to the minimal coupling hamiltonian can indeed produce the Power-Zienau-Woolley hamiltonian. Nevertheless, we demonstrate in this paper that both hamiltonians are not equivalent in the sense that they do not predict the same physical results. As we will show in the following, the Power-Zienau-Woolley hamiltonian creates photon states with a longitudinal polarization, *i*.*e*. non-physical photon states within the Coulomb gauge constraints. Here we should point out again that the phase-space is constrained. The constraints define a subspace (submanifold) where the dynamical variables are allowed to evolve. In order to make the physical results invariant through a unitary transformation, the unitary transformation must not modify the equations of constraints^36^ since only points on the phase-space submanifold represent physical states. However the Power-Zienau-Woolley transformation change the constraint satisfied by the vector potential as we demonstrate in the next paragraph.

Since a unitary transformation can not modify the commutators found in the Coulomb gauge (they are proportional to the identity operator), it can not implement a gauge transformation. The gauge being initially fixed to the Coulomb gauge, therefore after the unitary transformation the vector potential should satisfy again the Coulomb gauge condition. Nevertheless, if we have a look at the kinetic energy term of the Power-Zienau-Woolley hamiltonian given by the eq. (12), we conclude that the vector potential is ${\overrightarrow{A}}_{pzw}(\overrightarrow{x},t)=-\overrightarrow{x}\times {\int }_{0}^{1}\,\hat{B}(u\overrightarrow{x})udu$, an expression similar to eq. (5). Then it satisfies the Poincaré-gauge constraint $\overrightarrow{x}\mathrm{.}{\hat{A}}_{pzw}(\overrightarrow{x},t)=0$ instead of the Coulomb-gauge condition^1^. So the by Power *et al*.^1, 2, 4, 8–11^ has modify the equation of constraints without modifying the commutators correspondingly. A unitary transformation has to be applied with cares when a constrained hamiltonian is concerned since the dynamics is not defined in the entire phase-space but constrained on a subspace. As a consequence, we claim that the Power-Zienau-Woolley Hamiltonian is not unitary equivalent to the minimal-coupling hamiltonian. Up to our knowledge, these findings and the appropriate precautions inherent to a unitary transformation on the minimal-coupling hamiltonian are underestimated in the quantum-optics community since we have found no references, no textbooks mentioning them. Our result is not restricted to the case of the Power-Zienau-Woolley unitary transformation but extend to any unitary transformation applied to the minimal-coupling hamiltonian^9, 23, 37, 38^. It is not the goal of the present paper to determine if the unitary transformations used in the previously cited papers^9, 23, 37, 38^ change or not the equations of constraints but we want to emphasize that cautions should be taken in order to avoid generation of non-physical states.

^1^More details are given in the section (II-B) of the Supplementary Materials.

### The gauge is not fixed in the Power-Zienau-Woolley results

In order to show that the gauge is not properly fixed in the Power-Zienau-Woolley hamiltonian density, we need to write it in term of the electric and magnetic fields. To do so, we expand the momentum as ${\overrightarrow{\pi }}_{pwz}(\overrightarrow{x},t)={\varepsilon }_{0}{\overrightarrow{E}}^{\perp }(\overrightarrow{x},t)+{\overrightarrow{P}}^{\perp }(\overrightarrow{x},t)$. Then, the interaction term eq. (16) and the contact term eq. (17) cancel. Next, we remark that the self-interaction term eq. (15) is actually the contribution of the scalar potential in the Coulomb gauge (see [ref. 31, p. 373–378]) *i*.*e*. the contribution of the longitudinal-component of the electric field: eq. (15) = $\frac{1}{2}{\varepsilon }_{0}\overrightarrow{\nabla }{\varphi }_{c}{(\overrightarrow{x},t)}^{2}=\frac{1}{2}{\varepsilon }_{0}{\overrightarrow{E}}^{\parallel }{(\overrightarrow{x},t)}^{2}$ where ${\varphi }_{c}(\overrightarrow{x},t)$ is the scalar potential in the Coulomb gauge. The Helmholtz theorem states that any vector field is uniquely decomposed into transverse and longitudinal part. In such a case, whatever the gauge condition is, the transverse part of the electric field is given by ref. 39: ${\overrightarrow{E}}^{\perp }(\overrightarrow{x},t)=-{\partial }_{t}{\overrightarrow{A}}_{c}(\overrightarrow{x},t)$ where ${\overrightarrow{A}}_{c}(\overrightarrow{x},t)$ is the vector-potential in the Coulomb gauge. With the help of the eq. (5) that gives the expression of the vector potential in the Poincaré gauge, the Power-Zienau-Woolley Hamiltonian density finally reads:

$$\begin{array}{rcl}{ {\mathcal H} }_{pzw}(\overrightarrow{x},t) & = & {\xi }^{* }(\overrightarrow{x},t)\,\{(-\frac{{\hslash }^{2}}{2m})\,{[\overrightarrow{\nabla }-\frac{iq}{\hslash }{\overrightarrow{A}}_{p}(\overrightarrow{x},t)]}^{2}+V(\overrightarrow{x},t)\}\,\xi (\overrightarrow{x},t)\\  &  & +[\frac{1}{2}{\varepsilon }_{0}{\partial }_{t}{\overrightarrow{A}}_{c}^{\perp }{(\overrightarrow{x},t)}^{2}+\frac{1}{2}{\varepsilon }_{0}\overrightarrow{\nabla }{\varphi }_{c}{(\overrightarrow{x},t)}^{2}+\frac{1}{2{\mu }_{0}}{\overrightarrow{B}}^{2}(\overrightarrow{x},t)]\end{array}$$

The kinetic energy term is written in the Poincaré gauge whereas the electromagnetic-energy density is written in the Coulomb gauge. There is a gauge-inconsistency in the Power-Zienau-Woolley hamiltonian. In the quantum version, the operators algebra is undetermined since we do not know what is the gauge condition. So we do not know which commutators the operators have to satisfy.

We want to highlight that this observation is consistent with the derivation of the Power-Zienau-Woolley Hamiltonian density eqs (12–17) starting from a (semi-classical) lagrangian density^8^. In this reference, the authors broke the gauge symmetry of the lagrangian density by performing only the phase transformation of the wave function eq. (1) without performing the corresponding transformation of the gauge fields eqs (2) and (3). As a result the kinetic energy term is written in the new gauge (the Poincaré gauge) whereas the electromagnetic lagrangian density remains written in the initial gauge (the Coulomb gauge).

### The Power-Zienau-Woolley hamiltonian creates non-physical photon states

At the quantum level, the breaking of the gauge symmetry or said in other words, that the dynamical variables are no more constrained on the submanifold of physical states, has important consequences. The Power-Zienau-Woolley theory considers the vector and the scalar potentials in the Coulomb gauge as the dynamical variables^2, 7^. In this gauge the annihilation and creation operators generate transverse electromagnetic modes. The Fock space of photon states contains only transverse electromagnetic modes (*i*.*e*. photon-polarization states perpendicular to the wave-vector). On the opposite, as we derived in the Supplementary Materials (IV), the term ${\overrightarrow{A}}_{pzw}(\overrightarrow{x},t)=-\overrightarrow{x}\times {\int }_{0}^{1}\,\hat{B}(u\overrightarrow{x})udu$ generates photonic states with longitudinal component^2^ that do not belong to the Fock space. As a consequence, the Power-Zienau-Woolley hamiltonian generates photon states that do no satisfied the Coulomb gauge condition *i*.*e*. non-physical photon states. The contribution of the term ${\overrightarrow{A}}_{pzw}(\overrightarrow{x},t)$ is usually neglected^9, 23^ and, as far as we know, no references to the creation of non-physical photon states can be found in the literature.

## Discussion

To conclude, we have proved that the results obtained by Power *et al*. have some inconsistencies. The Power-Zienau-Woolley hamiltonian can not be derived from a gauge transformation since it mixes two gauge conditions. Even if a unitary transformation applied to the minimal-coupling hamiltonian generates the Power-Zienau-Woolley hamiltonian both results can not considered to be unitarily equivalent since the Power-Zienau-Woolley hamiltonian creates non-physical photon states. One can wonder why the inconsistencies of the Power-Zienau-Woolley hamiltonian have not been reported before our work. A possible explanation might hinge on the fact that most of the papers that use the Power-Zienau-Wooley hamiltonian perform one approximation before doing explicit calculations. Actually, all terms proportional to *P* ^⊥2^ are typically neglected. As a consequence, the self-interaction term eq. (15) is usually neglected and the interaction term reduced to ${\overrightarrow{P}}^{\perp }(\overrightarrow{x},t)\mathrm{.}\overrightarrow{E}(\overrightarrow{x},t)$. Within this approximation, the Power-Zienau-Woolley hamiltonian reduces to the minimal-coupling hamiltonian in the long-wavelength approximation. Consequencely, under some approximations, the Power-Zienau-Woolley Hamiltonian could be considered as a phenomenological hamiltonian. Nevertheless since it breaks the gauge symmetry it cannot be included in the framework of the well-established gauge theory^40^ that explains, as far as we know, all known experiments in quantum optics and in quantum electrodynamics but also many results in particle physics.

We have presented here a derivation of the multipolar hamiltonian starting from a lagrangian density. The hamiltonian density we have found depends only on the physical fields and may find applications in many fields of quantum physics. We have shown that in the multipolar gauge, there is no interaction term on the form $-\overrightarrow{P}(\overrightarrow{x},t\mathrm{).}\overrightarrow{D}(\overrightarrow{x},t)$. Instead, the interaction term is on the usual form ${\hat{\psi }}^{* }(\overrightarrow{x},t)\,\{(-\tfrac{{\hslash }^{2}}{2m})\,{[\overrightarrow{\nabla }-\tfrac{iq}{\hslash }A(\overrightarrow{x})]}^{2}\}\,\hat{\psi }(\overrightarrow{x},t)$. As a consequence, the *A* ^2^-term^23^ still occurs in the multipolar gauge. Highlighting that quantum electrodynamics is a constrained theory, we have shown that constraints between dynamical variables should be carefully taken into account otherwise some errors can occur. As far as we know, we give for the first time the correct commutators that apply in the Poincaré gauge. The properties of the Fock space in the Poincaré gauge will be the subject of a future paper.

## Electronic supplementary material

Supplementary Materials
