# Supplementary material for: The quantum-optics Hamiltonian in the Multipolar gauge
Source: Sci Rep. 2017 Sep 11;7:11115. doi: 10.1038/s41598-017-11076-5 (PMC5593841; doi:10.1038/s41598-017-11076-5)
Supplement: Supplementary file 1 — Supplementary Materials [file 41598_2017_11076_MOESM1_ESM.pdf]

The quantum-optics hamiltonian in the Multipolar gauge – Supplementary Materials

E. Rousseau, D. Felbacq
Université de Montpellier ,
Laboratoire Charles Coulomb UMR 5221,
F-34095, Montpellier, France

In this Supplementary Materials we detail the calculations useful to derive the quantum hamiltonian in the Poincaré (or multipolar) gauge. The commutation rules in this gauge are derived. We show that the correct dynamical equations are recovered with the help of the Heisenberg equation and the commutation rules. We start these Supplementary Materials by recalling some properties of gauge transformations in classical physics and in quantum physics. We highlight that the minimal-coupling hamiltonian in the Poincaré gauge is not equivalent to the Power-Zienau-Woolley hamiltonian neither through a gauge transformation nor through a unitary transformation.

Contents

I. Gauge transformation in classical physics: the hamiltonian in the multipolar gauge 1
A. Gauge transformation in the lagrangian formalism 2
B. Gauge transformation in the hamiltonian formalism 3
C. Precautions in deriving the hamiltonian from the lagrangian 4

II. Gauge transformation and unitary transformation at the quantum level 6
A. Gauge transformation of the Schrödinger equation 6
B. Unitary transformation of the vector-potential operator and of the kinetic-energy operator 7

III. Derivation of the commutation rules in the Poincaré gauge: The Dirac brackets in the Poincaré gauge 8
A. hamiltonian density 8
B. List of the constraints 9
C. The matrix of constraints **C** and its inverse **K** 10
D. The Dirac brackets 11
E. Commutation relations between quantum operators 13
F. Dynamical equations 13
1. The vector potential 14
2. The Schrödinger equation 14
3. The Maxwell-Ampère equation 15

IV. The Power-Zienau-Woolley hamiltonian creates non-physical states. 16

References 18

I. GAUGE TRANSFORMATION IN CLASSICAL PHYSICS: THE HAMILTONIAN IN THE MULTIPOLAR GAUGE

In the main article, we have considered all dynamical variables as fields (Schrödinger field or electromagnetic field). The equation of motion were obtained from a lagrangian density or a hamiltonian density. We can also consider a theoretical modeling where only the electromagnetic field variables are fields. The particle dynamical variables are its position and its momentum. In such a case, the equations of motion are obtained from a lagrangian function or from a hamiltonian function.

The goal of this section is to show that the Power-Zienau-Woolley hamiltonian does not derive from the minimal-coupling hamiltonian with the help of a gauge transformation on the opposite to the conclusion of the reference[1]. These results are reproduced in the textbook[2]. In order to explain the errors done in the reference[1], we first start to review some results about gauge transformation both in the lagrangian formalism and in the hamiltonian formalism. By writing the minimal-coupling hamiltonian in the multipolar gauge, we recover a result similar to the calculations

that we have done starting from a lagrangian density. We do not recover the Power-Zienau-Woolley hamiltonian on the opposite to ref[1]. The last subsection (I C) points out the main errors done in this reference[1].

A. Gauge transformation in the lagrangian formalism

In this section we review the main results about the minimal-coupling lagrangian and its transformation through a gauge transformation at the classical level. We consider a single particle with electric charge q in a binding potential $V(\vec{r})$. The minimal-coupling lagrangian in arbitrary gauge reads:

$$L(\vec{r},\vec{A},\phi) = \frac{1}{2}m\dot{\vec{r}}^{\,2} - V(\vec{r}) \tag{1}$$
$$+ \int d\vec{x}\frac{1}{2}\varepsilon_0[(\partial_t\vec{A}(\vec{x},t) + \nabla\phi(\vec{x},t))^2 - \frac{1}{2\mu_0}(\vec{\nabla}\times\vec{A}(\vec{x},t))^2] \tag{2}$$
$$+ q\dot{\vec{r}}.\vec{A}(\vec{r},t) - q\phi(\vec{r}) \tag{3}$$

The dynamical variables are the position of the particle $\vec{r}$, its velocity $\dot{\vec{r}}$, the electromagnetic-field degrees of freedom *i.e.* the vector-potential $\vec{A}(\vec{x},t)$ and the scalar potential $\phi(\vec{x},t)$. The first term eq:(1) is the lagrangian of the particle evolving in the binding potential $V(\vec{r})$. The second term eq:(2) is the lagrangian of the free electromagnetic-field and the third term eq:(3) describes the coupling between the particle and the electromagnetic-field. Note that in this interaction term, the electromagnetic potentials are evaluated at the particle position $\vec{r}$.

With the help of the Euler-Lagrange equations, this lagrangian ables to recover the dynamical equations of the particle given by the Newton law and the dynamical equations for the electromagnetic field given by the Maxwell equations. There are source terms given by a current density $\vec{j} = q\dot{\vec{r}}\delta(\vec{x}-\vec{r})$ and a charge density $\rho(\vec{x},t) = q\delta(\vec{x}-\vec{r})$.

We now fix the gauge. Depending on the condition we choose, the potentials satisfy either the Coulomb-gauge condition $\text{div}\vec{A}_c(\vec{x},t) = 0$ or the Poincaré-gauge condition $\vec{x}.\vec{A}_p(\vec{x},t)$. We denote $L_c(\vec{r},\vec{A}_c,\phi_c)$ the lagrangian written in the Coulomb gauge and $L_p(\vec{r},\vec{A}_p,\phi_p)$ the lagrangian written in the Poincaré gauge. At the classical level, one can pass from one gauge condition (*e.g.* the Coulomb gauge) to another (*e.g.* the Poincaré gauge) with the help of a gauge-generating function $\chi_{c\to p}(\vec{x},t)$.

$$\{\vec{A}_c(\vec{x},t),\phi_c(\vec{x},t)\} \xrightarrow{\chi_{c\to p}(\vec{x},t)} \{\vec{A}_p(\vec{x},t),\phi_p(\vec{x},t)\}$$

Starting from the Coulomb gauge, the electromagnetic potentials in the Poincaré gauge are given by[3]:

$$\vec{A}_p(\vec{x},t) = \vec{A}_c(\vec{x},t) + \vec{\nabla}\chi_{c\to p}(\vec{x},t)$$
$$\phi_p(\vec{x},t) = \phi_c(\vec{x},t) - \partial_t\chi_{c\to p}(\vec{x},t)$$

Now we replace these expressions in $L_c(\vec{r},\vec{A}_c,\phi_c)$:

$$\begin{aligned} L_c(\vec{r},\vec{A}_c,\phi_c) &= \frac{1}{2}m\dot{\vec{r}}^{\,2} - V(\vec{r}) \\ &+ \int d\vec{x}\frac{1}{2}\varepsilon_0[(\partial_t\vec{A}_p(\vec{x},t) + \nabla\phi_p(\vec{x},t))^2 - \frac{1}{2\mu_0}(\vec{\nabla}\times\vec{A}_p(\vec{x},t))^2] \\ &+ q\dot{\vec{r}}\,.\,(\vec{A}_p(\vec{r},t) - \vec{\nabla}\chi_{c\to p}(\vec{r},t)) - q(\phi_p(\vec{r}) + \partial_t\chi_{c\to p}(\vec{r},t)) \end{aligned}$$

We can rewrite the interaction term and the Lagrangian function reads:

$$\begin{aligned} L_c(\vec{r},\vec{A}_c,\phi_c) &= \frac{1}{2}m\dot{\vec{r}}^{\,2} - V(\vec{r}) \\ &+ \int d\vec{x}\frac{1}{2}\varepsilon_0[(\partial_t\vec{A}_p(\vec{x},t) + \nabla\phi_p(\vec{x},t))^2 - \frac{1}{2\mu_0}(\vec{\nabla}\times\vec{A}_p(\vec{x},t))^2] \\ &+ q\dot{\vec{r}}\,.\,\vec{A}_p(\vec{r},t) - q\phi_p(\vec{r}) \\ &- q[\dot{\vec{r}}\,.\,\vec{\nabla}\chi_{c\to p}(\vec{r},t) + \partial_t\chi_{c\to p}(\vec{r},t)] \end{aligned}$$

The last term is actually the total time-derivative of the gauge-generating function since the particle position $\vec{r}$ depends on the time: $\frac{d}{dt}\chi_{c\to p}(\vec{r},t) = \partial_t \chi_{c\to p}(\vec{r},t) + \dot{\vec{r}}\ .\ \vec{\nabla}\chi_{c\to p}(\vec{r},t)$.

Defining, the lagrangian in the Poincaré gauge, $L_p(\vec{r}, A_p, \phi_p)$, as:

$$\begin{aligned} L_p(\vec{r}, \vec{A}_p, \phi_p) &= \frac{1}{2} m \dot{\vec{r}}^{\,2} - V(\vec{r}) \\ &+ \int d\vec{x} \frac{1}{2} \varepsilon_0 [(\partial_t \vec{A}_p(\vec{x},t) + \nabla \phi_p(\vec{x},t))^2 - \frac{1}{2\mu_0} (\vec{\nabla} \times \vec{A}_p(\vec{x},t))^2] \\ &+ q\dot{\vec{r}}\ .\ \vec{A}_p(\vec{r},t) - q\phi_p(\vec{r}) \end{aligned}$$

One can relate the lagrangian written in the Coulomb gauge to the lagrangian written in the Poincaré gauge through the relationship:

$$L_p(\vec{r}, \vec{A}_p, \phi_p) = L_c(\vec{r}, \vec{A}_c, \phi_c) + \frac{d}{dt}[q\chi_{c\to p}(\vec{r},t))] \tag{4}$$

Adding a total time-derivative does not change the Euler-Lagrange equations since the action remains extremal. So the two lagrangians $L_c(\vec{r}, \vec{A}_c, \phi_c)$ and $L_p(\vec{r}, \vec{A}_p, \phi_p)$ are *equivalent* starting point to derive the dynamical equations. The gauge transformation is then a canonical transformation[4] *i.e.* a change of variables. It even preserves the form of the lagrangian (and of the hamiltonian as we will show in the following). The lagrangian or the hamiltonian in the new gauge are simply obtained by substituting the old coordinates by the new ones. As a consequence, in the Coulomb gauge, $\vec{A}_c$ and ϕ_c are the dynamical variables whereas $\vec{A}_p$ and ϕ_p are the dynamical variables in the Poincaré gauge. As a result, the canonical-momentum conjugated to the potential vector is gauge independent. Indeed, in the Coulomb gauge, it is calculated as:

$$\vec{\pi}_c(\vec{x},t) = \frac{\partial L_c}{\partial(\partial_t A_c)}(\vec{r}, \vec{A}_c, \phi_c) = \varepsilon_0[\partial_t \vec{A}_c(\vec{x},t) + \vec{\nabla}\phi_c(\vec{x},t)] = -\varepsilon_0 \vec{E}(\vec{x},t) \tag{5}$$

And in the Poincaré gauge it reads:

$$\vec{\pi}_p(\vec{x},t) = \frac{\partial L_p}{\partial(\partial_t A_p)}(\vec{r}, \vec{A}_p, \phi_p) = \varepsilon_0[\partial_t \vec{A}_p(\vec{x},t) + \vec{\nabla}\phi_p(\vec{x},t)] = -\varepsilon_0 \vec{E}(\vec{x},t)$$

The canonical-momentum conjugated to the vector potential is equal to the electric field times "$-\varepsilon_0$" independently of the choice of gauge. This is in agreement with the gauge invariance of the electric field whereas ref[1] has reached an opposite conclusion (see subsection (I C) for more details).

B. Gauge transformation in the hamiltonian formalism

In this subsection, we first convert the lagrangian function to the hamiltonian function from a Legendre transformation. Next we show that the hamiltonian in the Poincaré gauge is not the Power-Zienau-Woolley hamiltonian in the opposite to the conclusion of the ref.[1, 2].

In order to compute the hamiltonian, we need to know the canonical momentum conjugated to the particle position $\vec{r}$. In the Coulomb gauge it reads:

$$\vec{\mathcal{P}} = \frac{\partial L_c}{\partial \dot{r}}(\vec{r}, \vec{A}_c, \phi_c) = m\dot{\vec{r}} + q\vec{A}_c(\vec{r},t) \tag{6}$$

and in the Poincaré gauge,

$$\vec{\mathcal{P}} = \frac{\partial L_p}{\partial \dot{r}}(\vec{r}, \vec{A}_p, \phi_p) = m\dot{\vec{r}} + q\vec{A}_p(\vec{r},t) \tag{7}$$

Note that the canonical conjugate momentum $\vec{\mathcal{P}}$ depends on the gauge through the vector potential $\vec{A}_c(\vec{r},t) \neq \vec{A}_p(\vec{r},t)$. The Legendre transformation converts the lagrangian function to the hamiltonian function:

$$H(\vec{r},\vec{p},\vec{A},\vec{\pi}) = \vec{\mathcal{P}}.\dot{\vec{r}} + \int d\vec{x}\vec{\pi}(\vec{x},t).\partial_t\vec{A}(\vec{x},t) - L(\vec{r},\vec{A})$$
$$H(\vec{r},\vec{p},\vec{A},\vec{\pi}) = \frac{1}{2m}[\vec{\mathcal{P}} - q\vec{A}(\vec{r},t)]^2 + V(\vec{r}) + \int d\vec{x}[\frac{1}{2\varepsilon_0}\vec{\pi}(\vec{x},t)^2 + \frac{1}{2\mu_0}(\vec{\nabla}\times\vec{A}(\vec{x},t))^2]$$

Obviously, in the Coulomb gauge, we find:

$$H_c(\vec{r},\vec{p},\vec{A}_c,\vec{\pi}_c) = \frac{1}{2m}[\vec{\mathcal{P}} - q\vec{A}_c(\vec{r},t)]^2 + V(\vec{r}) + \int d\vec{x}[\frac{1}{2\varepsilon_0}\vec{\pi}_c(\vec{x},t)^2 + \frac{1}{2\mu_0}(\vec{\nabla}\times\vec{A}_c(\vec{x},t))^2]$$

And, in the Poincaré gauge, the minimal-coupling hamiltonian reads:

$$H_p(\vec{r},\vec{p},\vec{A}_p,\vec{\pi}_p) = \frac{1}{2m}[\vec{\mathcal{P}} - q\vec{A}_p(\vec{r},t)]^2 + V(\vec{r}) + \int d\vec{x}[\frac{1}{2\varepsilon_0}\vec{\pi}_p(\vec{x},t)^2 + \frac{1}{2\mu_0}(\vec{\nabla}\times\vec{A}_p(\vec{x},t))^2]$$

In the Poincaré gauge, the potentials can be written with the physical fields,

$$H_p(\vec{r},\vec{p},\vec{A}_p,\vec{\pi}_p) = \frac{1}{2m}[\vec{\mathcal{P}} + q\vec{r}\times\int_0^1 udu\vec{B}(u\vec{r},t)]^2 + V(\vec{r}) + \int d\vec{x}[\frac{1}{2}\varepsilon_0\vec{E}(\vec{x},t)^2 + \frac{1}{2\mu_0}B^2(\vec{x},t)]$$

Where we have used the link between the vector potential in the Poincaré gauge $\vec{A}_p$ and the magnetic field $\vec{B}$ [3]: $\vec{A}_p(\vec{x},t) = -\vec{r}\times\int_0^1 udu\vec{B}(u\vec{r},t)$. This result is similar to the results we have obtained in the main paper starting from a lagrangian density.

We want to make three important remarks here:

- Firstly, the kinetic energy term $\mathcal{K} = \frac{1}{2m}[\vec{\mathcal{P}} + q\vec{r}\times\int_0^1 udu\vec{B}(u\vec{r},t)]^2$ reads similarly in our result and in the Power-Zienau-Woolley hamiltonian meaning that in both results the potential vector is $\vec{A}_p(\vec{x},t) = -\vec{r}\times\int_0^1 udu\vec{B}(u\vec{r},t)$. As a consequence, the vector potential in the Power-Zienau-Woolley hamiltonian satisfies the Poincaré gauge condition $\vec{x}.\vec{A}_p(\vec{x},t) = 0$. This is not the conclusion of previous authors[1, 2, 5–12] for which the vector potential satisfies the Coulomb gauge conditions.

- Secondly, on the opposite to the Power-Zienau-Woolley hamiltonian, there is no coupling term on the form $-\frac{1}{\varepsilon_0}\int d\vec{x}\vec{D}(\vec{x},t).\vec{P}(\vec{x},t)$ where $\vec{D}(\vec{x},t)$ is the displacement vector and $\vec{P}(\vec{x},t)$ is the polarization field. There is also no contact term on the form $\frac{1}{2\varepsilon_0}\int d\vec{x}\vec{P}(\vec{x},t)^2$

- Thirdly, note the important point that the electromagnetic energy term remains equal to $\int d\vec{x}[\frac{1}{2}\varepsilon_0\vec{E}(\vec{x},t)^2 + \frac{1}{2\mu_0}B^2(\vec{x},t)]$, independently of the gauge. This the electromagnetic field energy in vacuum. In the Power-Zienau-Wooolley hamiltonian, previous authors [1, 2, 5–12] have found that the electromagnetic-field energy term reads $\int d\vec{x}[\frac{1}{2\varepsilon_0}\vec{D}(\vec{x},t)^2 + \frac{1}{2\mu_0}B^2(\vec{x},t)]$. The physical interpretation of this expression is not straightforward since it is neither the electromagnetic energy in vacuum nor in matter[3]. The following section explains in more details the errors that lead to this inconsistent result.

C. Precautions in deriving the hamiltonian from the lagrangian

In the reference [1], Babiker and London derived the Power-Zienau-Woolley hamiltonian from a gauge transformation performed on the lagrangian. Starting from the minimal-coupling lagrangian in the Coulomb gauge, they added the total derivative of the gauge-generating function $\chi(\vec{r}) = -\int_0^1 \vec{r}.\vec{A}_c(u\vec{r})du$ [3]. This gauge-generating function ables to pass from the Coulomb gauge to the Poincaré gauge[3]. The starting point used by Babiker and Loudon is the eq:(4) of these Supplementary Materials. As explained previously this equation performs a gauge transformation but does not modify the equations of motion:

$$
\begin{aligned}
L'(\vec{r},\vec{A}_c,\phi_c) &= L(\vec{r},\vec{A}_c,\phi_c) - q\frac{d}{dt}[\vec{r}.\int_0^1 \vec{A}_c(u\vec{r})du] \\
&= L(\vec{r},\vec{A}_c,\phi_c) - q\dot{\vec{r}}.\int_0^1 \vec{A}_c(u\vec{r})du - q\vec{r}.\int_0^1 \partial_t\vec{A}_c(u\vec{r})du - q\vec{r}.\int_0^1 (u\dot{\vec{r}}.\vec{\nabla}_{u\vec{r}})\vec{A}_c(u\vec{r})du \qquad (8)
\end{aligned}
$$

We want to emphasize here that *despite the gauge transformation* they considered the potentials in the Coulomb gauge $\vec{A}_c$ and ϕ_c as being the dynamical variables.

Then, they have computed the canonical momentum $\vec{\pi}$ conjugated to the vector potential $\vec{A}_c$. They have found with the help of equation eq:(8):

$$
\begin{aligned}
\vec{\pi}'(\vec{x},t) &= \frac{\partial\mathcal{L}'[\vec{A}_c,\partial_t\vec{A}_c]}{\partial\partial_t\vec{A}_c(\vec{x})} \\
&= \varepsilon_0[\partial_t\vec{A}_c(\vec{x},t) + \vec{\nabla}\phi_c(\vec{x},t)] - q\vec{r}\int_0^1 \delta(\vec{r}-u\vec{x})du \\
&= -\vec{D}(\vec{x},t) \qquad (9)
\end{aligned}
$$

The first term is the electric field up to a constant $\varepsilon_0[\partial_t\vec{A}_c(\vec{x},t) + \vec{\nabla}\phi_c(\vec{x},t)] = -\varepsilon_0\vec{E}(\vec{x},t)$ and the second term is assumed to be the polarization field defined as $\vec{P}(\vec{x},t) = q\vec{r}\int_0^1 \delta(\vec{r}-u\vec{x})du$. This definition is said to embody the contribution of all the electric multipoles moments [6] (see also ref.[12, p.318] and ref.[2, p.283]). The important conclusion to previous authors[1, 2] is that the canonical momentum $\vec{\pi}'(\vec{x},t)$ is identified to the opposite of the displacement vector $\vec{D}(\vec{x},t)$.

We think that this calculation is incomplete and that the authors of ref.[1, 2] have made some errors.

1. First, they did not realize that a gauge transformation is also a canonical transformation [4]. This means that a gauge transformation is a change of variables. In such a case, it is not correct to consider that the potentials in the Coulomb gauge remain the dynamical variables in the Poincaré gauge.

2. Secondly, even if they did not notice the change of dynamical variables, the canonical momentum conjugated to the vector potential does not satisfy the constraints that act on the hamiltonian. Indeed, as we explain in details later on, several constraints act to the {field+particle}-dynamics. One of them is the absence of canonical momentum to the scalar potential $\pi_\phi = 0$. This constraint has to hold at any time. It induces the following constraint $\vec{\nabla}.\vec{\pi} = -q\delta(\vec{x}-\vec{r})$, which is nothing else but the Maxwell-Gauss equation. When calculating the canonical momentum to the vector potential, the functional derivative with respect to $\partial_t\vec{A}$ is not well defined because the vector potential satisfies the gauge constraints (see ref.[13, p.348]). We reproduce here the reasoning of this book. We fix the gauge to be the Coulomb gauge. Coming back to the definition of the functional derivative with respect to $\partial_t\vec{A}_c$, we can write that any variations of $\delta[\partial_t\vec{A}_c]$ induce a variation of the lagrangian given by:

$$
\delta L' = \int d\vec{x}\frac{\partial\mathcal{L}'}{\partial\partial_t\vec{A}_c}.\delta[\partial_t\vec{A}_c]
$$

In the Coulomb gauge, the vector potential satisfies: $\vec{\nabla}.\vec{A}_c(\vec{x},t) = 0$. Then, without changing the variations of the lagrangian, one can add the gradient of any scalar function. Indeed,

$$
\begin{aligned}
\delta L' &= \int d\vec{x}(\frac{\partial\mathcal{L}'}{\partial\partial_t\vec{A}_c} + \vec{\nabla}\mathcal{F}(\vec{x})).\delta[\partial_t\vec{A}_c] \\
&= \int d\vec{x}(\frac{\partial\mathcal{L}'}{\partial\partial_t\vec{A}_c}).\delta[\partial_t\vec{A}_c] - \int d\vec{x}\mathcal{F}(\vec{x})\vec{\nabla}.\delta[\partial_t\vec{A}_c] \\
&= \int d\vec{x}(\frac{\partial\mathcal{L}'}{\partial\partial_t\vec{A}_c}).\delta[\partial_t\vec{A}_c] \qquad (10)
\end{aligned}
$$

The second line is obtained after an integration by part. The third line follows from the Coulomb gauge condition $\vec{\nabla}.\vec{A}_c = 0$ implying that functional variations of the vector potential have to satisfy the Coulomb-gauge conditions as well. So $\vec{A}_c \to \vec{A}_c + \delta\vec{A}_c$ implies that $\vec{\nabla}.(\vec{A}_c + \delta\vec{A}_c) = 0 \to \vec{\nabla}.(\delta\vec{A}_c) = 0$ since $\vec{\nabla}.\vec{A}_c = 0$. Hence, we conclude that $\vec{\nabla}.\delta[\partial_t\vec{A}_c] = 0$.

To summarize, following ref.[13], the conjugated momentum $\vec{\pi}'$ is computed from the functional derivative up to the gradient of a scalar function (in the Coulomb-gauge). This degree of freedom is fixed by the Maxwell-Gauss equation.

The canonical momentum of the vector potential calculated by Babiker and Loudon $\vec{\pi}'(\vec{x},t)$, *i.e.* eq:(9), does not satisfy the constraint $\vec{\nabla}.\vec{\pi}'(\vec{x},t) = -q\delta(\vec{x}-\vec{r})$ since from its definition [eq:(9)] and the Maxwell-Gauss equation, one should have $\vec{\nabla}.\vec{\pi}'(\vec{x},t) = \vec{\nabla}.\vec{D}(\vec{x},t) = 0$. Nevertheless, as just explained above, we are free to add the gradient of any scalar function in order to recover the Maxwell-Gauss equation. Then we can choose to add $\vec{\nabla}\mathcal{F}(\vec{x}) = q\vec{r}\int_0^1 \delta(\vec{r}-u\vec{x})du$ to $\vec{\pi}'(\vec{x},t)$ without changing the lagrangian-variations $\delta L'$:

$$\vec{\pi}''(\vec{x},t) = \vec{\pi}'(\vec{x},t) + q\vec{r}\int_0^1 \delta(\vec{r}-u\vec{x})du$$

Finally, the canonical momentum of the vector potential that satisfies the constraint $\vec{\nabla}.\vec{\pi}''(\vec{x},t) = -q\delta(\vec{x}-\vec{r})$ reads:

$$\vec{\pi}''(\vec{x},t) = \varepsilon_0[\partial_t\vec{A}(\vec{x},t) + \vec{\nabla}\phi(\vec{x},t)]$$

This expressions is in agreement with our previous calculation [eq:(6)] and with the gauge invariance of the electric field (it remains $\varepsilon_0[\partial_t\vec{A}(\vec{x},t)+\vec{\nabla}\phi(\vec{x},t)]$). The derivation of the Power-Zienau-Woolley hamiltonian requires $\vec{\pi}'(\vec{x},t) = -\vec{D}(\vec{x},t)$[1, 2] that is not compatible with the gauge invariance of electromagnetism. So we conclude that the Power-Zienau-Woolley hamiltonian cannot be obtained with the help of a gauge transformation.

II. GAUGE TRANSFORMATION AND UNITARY TRANSFORMATION AT THE QUANTUM LEVEL

Historically, the Power-Zienau-Woolley hamiltonian has been derived with the help of a unitary transformation applied to the minimal-coupling hamiltonian written in the Coulomb gauge[5]. Even if this can always be done, we will show in the following of these supplementary materials that some cares are required because electrodynamics is a constraint theory. The unitary transformation should leave invariant the equations of constraints. This is not the case for the Power-Zienau-Woolley unitary transformation. As a consequence, as we will show in the following, the Power-Zienau-Wolley hamiltonian predicts unphysical photon-states (see section (IV) for more details).

A. Gauge transformation of the Schrödinger equation

At the quantum level, the gauge invariance manifests itself by a transformation of the wavefunction through a unitary transformation. In order to get the measurements independent of the gauge conditions, the hamiltonian has to be modified as well [14]:

$$\psi_p(\vec{x},t) = \exp[i\frac{q}{\hbar}\chi(\vec{r},\vec{A}_c,t)]\psi_c(\vec{x},t) \tag{11}$$

$$\hat{H}_p = \exp[i\frac{q}{\hbar}\chi(\vec{r},\vec{A}_c,t)]\hat{H}_c\exp[-i\frac{q}{\hbar}\chi(\vec{r},\vec{A}_c,t)] - \exp[i\frac{q}{\hbar}\chi(\vec{r},\vec{A}_c,t)]\frac{\partial}{\partial t}\exp[-i\frac{q}{\hbar}\chi(\vec{r},\vec{A}_c,t)] \tag{12}$$

The gauge-transformation of the Schrödinger equation has been first exhibited by Weyl ([15] pp.100-101). It corresponds to the multiplication of the wavefunction by a phase factor which is actually a unitary operator. Keep in mind that the unitary operator $\hat{U} = \exp[i\frac{q}{\hbar}\chi(\vec{r},\vec{A}_c,t)]$ changes the vector potential and the scalar potential from one gauge to another.

At the quantum-field level, when both the Schrödinger field $\hat{\psi}(\vec{x},t)$ and the electromagnetic field $\{\hat{A}(\vec{x},t),\hat{\phi}(\vec{x},t)\}$are considered as operators, the equivalent of equation (12) does not exist (p.70 in [16]). One has to fix the gauge at the classical level and then quantize the theory. As a consequence, one cannot change the gauge on the (quantum) minimal-coupling hamiltonian by applying the eq:(12).

B. Unitary transformation of the vector-potential operator and of the kinetic-energy operator

In this paragraph, we examine some details about the derivation of the Power-Zienau-Woolley hamiltonian from a unitary transformation. We highlight that (i) a unitary transformation can change the potential vector operator, the gauge transformation at the quantum level is indeed a unitary transformation (ii) as the unitary transformation does not change the commutators (they are proportional to the identity operator in the Coulomb gauge), if the vector potential operator is changed by the unitary transformation, it may satisfy some constraints that are no more reflected by the commutators. As a consequence, cares are required when applying a unitary transformation to the quantum minimal-coupling hamiltonian. The unitary transformation should not modify the equations of constraint satisfied by the vector potential and the scalar potential. In the opposite case, if they are changed by the unitary transformation, the new constraints are not taken into account by the commutators. This important point has not been recognized by the previous authors[1, 2, 5, 7–10, 12, 17] since we will show that the unitary transformation used by Power *et al.* indeed modifies the vector potential.

Let us illustrate our claims. Through a unitary transformation of the Schrödinger equation, the initial wavefunction $|\psi\rangle_i$and the hamiltonian $\hat{H}_i$ transform as:

$$\begin{aligned} |\psi\rangle_f &= \hat{U}\,|\psi\rangle_i \\ \hat{H}_f &= \hat{U}\hat{H}_i\hat{U}^\dagger - \hat{U}\frac{d}{dt}\hat{U}^\dagger \end{aligned} \tag{13}$$

Of course there are similarities between these equations [eq:(13)] and the equations of the gauge transformation of the Schrödinger equation [eq:(12)]. Again, both the wavefunction and the hamiltonian have to be modified in order to keep the measurements independent of the unitary transformation.

Power and coworkers extended the validity of the equation (13) to the case of the minimal-coupling hamiltonian that describes both the particle and the electromagnetic field dynamics. Moreover they do not take into account the contribution of $\hat{U}\frac{d}{dt}\hat{U}^\dagger$ because the operator U they considered does not depend explicitly on time $\frac{\partial}{\partial t}\hat{U}$ (see *e.g.* the introduction of ref. [10]).

They started from the Coulomb gauge, the dynamical variables being the vector-potential operator $\hat{A}_c(\vec{x},t)$ and the transverse part of its canonical momentum $\hat{\pi}_c^\perp(\vec{x},t)$. As a consequence, the minimal-coupling hamiltonian operator $\hat{H}_c^{mc}$ and the canonical commutation relations satisfied by the field operators read[14]:

$$\hat{H}_c^{mc} = \frac{1}{2m}[\hat{\mathcal{P}} - q\hat{A}_c(\hat{r},t)]^2 + V(\hat{r}) + \int d\vec{x}\left\{\frac{1}{2\varepsilon_0}\hat{\pi}_c^\perp(\vec{x},t)^2 + \frac{1}{2\mu_0}\left[\vec{\nabla}\times\hat{A}_c(\vec{x},t)\right]^2\right\} \tag{14}$$

$$\hat{\pi}_c^\perp(\vec{x},t) = -\varepsilon_0\hat{E}_c^\perp(\vec{x},t) \tag{15}$$

$$[\hat{A}_c^i(\vec{x},t), \hat{E}_j^\perp(\vec{y},t)] = -\frac{i\hbar}{\varepsilon_0}\delta_{i,j}^T(\vec{x}-\vec{y})\mathbf{1} \tag{16}$$

where $i,j = 1,2,3$ and $\mathbf{1}$ is the identity matrix. This minimal-coupling hamiltonian eq:(14) described a single electron evolving in the classical potential $V(\hat{r})$ (*e.g.* created by the nucleus of the atom) and in interaction with a quantum electromagnetic-field. The last relationship eq:(16) involves the transverse Dirac-distribution defined as

$$\delta_{i,j}^T(\vec{x}-\vec{y}) = \delta_j^i\delta(\vec{x}-\vec{y}) - \frac{\partial^2}{\partial x_i\partial y_j}\frac{1}{4\pi|\vec{x}-\vec{y}|}$$

where δ_j^i is the Kroeneker symbol (see *e.g.* ref.[2] p. 38). The transverse Dirac-distribution selects the transverse component of a vector function.

As a unitary transformation, Power *al.*[1, 2, 5, 17] have chosen $\hat{U} = \exp\left[i\frac{q}{\hbar}\hat{\chi}(\hat{r},\hat{A}_c,t)\right]$ with $\hat{\chi}(\hat{r},\hat{A}_c,t) = -\int_0^1 \hat{r}.\hat{A}_c(u\vec{x},t)du$. Note that this last operator $\hat{\chi}(\hat{r},\hat{A}_c,t)$ is similar to the gauge-generating function of the Poincaré gauge[3].

Then Power *et al.* [1, 2, 5, 17] transform the minimal-coupling hamiltonian operator $\hat{H}_c^{mc}$ with the help of the unitary transformation $\hat{U}$.

This is done with the help of the Campbell-Baker-Hausdorff formula:

$$\exp(-\hat{S})\hat{O}\exp(\hat{S}) = \hat{O} - [\hat{S},\hat{O}] + \frac{1}{2!}[\hat{S},[\hat{S},\hat{O}]] + ...$$

With $S = -i\frac{q}{\hbar}\chi(\vec{r}, \vec{A}_c, t)$ and recalling that in any gauge, the canonical variables $\{\hat{r}, \hat{\mathcal{P}}\}$ satisfied the canonical commutation relations[18] $[\hat{r}_\mu, \hat{\mathcal{P}}_\nu] = i\hbar\delta_{\mu,\nu}$, Power *et al.* [1, 2, 5, 17] have found the following results:

$$\hat{U}\hat{r}\hat{U}^\dagger = \hat{r} \tag{17a}$$

$$\hat{U}\hat{A}_c\hat{U}^\dagger = \hat{A}_c(\hat{r}, t) \tag{17b}$$

$$\hat{U}\hat{\mathcal{P}}\hat{U}^\dagger = \hat{\mathcal{P}} - q\vec{\nabla}\hat{\chi}(\hat{r}, \hat{A}_c, t) \tag{17c}$$

$$\hat{U}[\hat{A}_c^i(\vec{x}, t), \hat{E}_j^\perp(\vec{y}, t)]\hat{U}^\dagger = -\frac{i\hbar}{\varepsilon_0}\delta_{i,j}^T(\vec{x} - \vec{y})\hat{U}\mathbf{1}\hat{U}^\dagger = -\frac{i\hbar}{\varepsilon_0}\delta_{i,j}^T(\vec{x} - \vec{y})\mathbf{1} \tag{17d}$$

Note that the unitary transformation does not change the canonical commutation relation between the field operators [eq:(17d)].

Equations eq:(17b) and eq:(17c) help us to conclude that the linear momentum transforms as

$$\begin{aligned} \hat{U}^\dagger(\hat{\mathcal{P}} - q\hat{A}_c)\hat{U} &= \hat{\mathcal{P}} - q\vec{\nabla}\hat{\chi}(\hat{r}, \hat{A}_c, t) - q\hat{A}_c \\ &= \hat{\mathcal{P}} - q\hat{A}_p \end{aligned} \tag{18}$$

Here we have recognize the vector potential in the Poincaré gauge[3]: $\hat{A}_p(\hat{r}, t) = \hat{A}_c(\hat{r}, t) + \vec{\nabla}\chi(\hat{r}, t)$.

The analytical expression of the linear momentum ables to conclude that the potential vector after the unitary transformation is $\hat{A}_p$, the vector potential in the Poincaré gauge.

On the opposite, the previous authors have misinterpreted the eq:(17b) and they have concluded that the unitary transformation does not change the vector potential. This is wrong since the quantity of physical interest is the linear momentum of the particle position $m\dot{\hat{r}}$. Indeed, it transforms under the unitary transformation as the corresponding classical-quantity does through a gauge transformation[19]. As a consequence, the potential vector operator must be extracted from the equation (18) and not from the equation (17b). Obviously $\hat{A}_p(\hat{r}, t)$ doesn't satisfy the canonical commutation relations eq:(17d). This important conclusion has been missed by the previous authors[1, 2, 5, 8–10, 17]. Because quantum electrodynamics is a constrained theory, cares should be taken before applying a unitary transformation to the minimal-coupling hamiltonian. One should check that the unitary transformation does not modify the constraints between the dynamical variables. The most important consequence is that the Power-Zienau-Woolley hamiltonian produces unphysical results. For example, it leads to the creation of longitudinal photons in the Coulomb gauge (see section (IV)). This remark leads to the important conclusion that the Power-Zienau-Woolley hamiltonian cannot be considered to be unitarily equivalent to the minimal-coupling hamiltonian. Indeed, in phase-space, the unitary transformation does not leave invariant the sub-manifold of physical states.

III. DERIVATION OF THE COMMUTATION RULES IN THE POINCARÉ GAUGE: THE DIRAC BRACKETS IN THE POINCARÉ GAUGE

A. hamiltonian density

Following the notation used in the main article, we use the Einstein summation convention. Repeated indexes are summed but we discriminate the time variable since we are working in a non-relativistic approximation. ϕ and ψ are respectively the scalar potential and the Schrödinger field. As a consequence, π_ϕ and π_ψ are the canonical momenta conjugated respectively to ϕ and ψ . In the Dirac theory, the constraints acting on the dynamics applied effectively at the end of the calculation. As a consequence the hamiltonian density derived from a lagrangian density reads:

$$\begin{aligned} \mathcal{H}(\vec{x}, t) &= \frac{\hat{\pi}_\psi^\dagger(\vec{x}, t)}{i\hbar}\{-\frac{\hbar^2}{2m}[\partial_\mu - \frac{iq}{\hbar}\hat{A}_\mu(\vec{x}, t)]^2 + V(\vec{x}, t)\}\hat{\psi}(\vec{x}, t) + \\ &\quad \frac{1}{2\varepsilon_0}\hat{\pi}^\mu(\vec{x}, t)\hat{\pi}_\mu(\vec{x}, t) + \frac{1}{2\mu_0}\hat{B}^\mu(\vec{x}, t)\hat{B}_\mu(\vec{x}, t) + \\ &\quad [\partial_\mu\pi^\mu(\vec{x}, t) + \frac{q}{i\hbar}\pi_\psi(\vec{x}, t)\psi(\vec{x}, t)]\phi(\vec{x}, t) \end{aligned}$$

This expression follows after having assuming that all fields vanish at infinities. The last term, which is the Maxwell-Gauss equation, is not taken to be zero at the beginning of the calculations. Only at the end, after the computation of the Dirac brackets, it will be assumed to be null.

B. List of the constraints

We now enumerate the constraints that act on the dynamics of the system constituted by the particle and the electromagnetic field. Four constraints acts on the dynamics of this system. These constraints defines a sub-manifold by restricting the available phase-space spanned by the 5 couples of conjugated variables. They are: $(\psi, \pi_\psi), (\phi, \pi_\phi), (A^\mu, \pi_\mu)_{\mu=1,2,3}$

1- The first constraint is the absence of momentum conjugated to the scalar potential $\phi(\vec{x}, t)$ since there is no term proportional to $\partial_t \phi(\vec{x}, t)$ in the lagrangian density. As a consequence, the canonical momentum $\pi_\phi(\vec{x}, t)$ is identically null. In the Dirac theory[20], $\pi_\phi(\vec{x}, t) = 0$ appears as one constraint that restricts the available phase-space. We note $\chi_1(\vec{x}, t)$ this first constraint:

$$\chi_1(\vec{x}, t) = \pi_\phi(\vec{x}, t)$$

2- The Maxwell-Gauss equation is the second constraint. It derives from the previous constraint.

Indeed, the previous constraint has to remain true at any time, so $\dot{\chi}_1(\vec{x}, t) = 0$. The time derivative of $\chi_1(\vec{x}, t)$ is computed with the help of Poisson brackets defined as:

$$\begin{aligned}
\{F, G\}(\vec{x}, t) &= \frac{\delta F}{\delta \psi} \frac{\delta G}{\delta \pi_\psi} - \frac{\delta G}{\delta \psi} \frac{\delta F}{\delta \pi_\psi} \\
&+ \frac{\delta F}{\delta \phi} \frac{\delta G}{\delta \pi_\phi} - \frac{\delta G}{\delta \phi} \frac{\delta F}{\delta \pi_\phi} \\
&+ \frac{\delta F}{\delta A^\mu} \frac{\delta G}{\delta \pi_\mu} - \frac{\delta G}{\delta A^\mu} \frac{\delta F}{\delta \pi_\mu}
\end{aligned}$$

Where $\frac{\delta F}{\delta \phi} = \frac{\partial F}{\partial \phi} - \partial_\nu \frac{\partial F}{\partial_\nu \phi}$ is the functional derivative (or Frêchet derivative).
We have found as a second constraint:

$$\begin{aligned}
\chi_2(\vec{x}, t) &= \{\chi_1, \mathcal{H}\}(\vec{x}, t) = -\frac{\delta \mathcal{H}}{\delta \phi} \frac{\delta \chi_1}{\delta \pi_\phi} \\
&= -[\partial_\mu \pi^\mu(\vec{x}, t) + \frac{q}{i\hbar} \pi_\psi(\vec{x}, t) \psi(\vec{x}, t)] \\
\chi_2(\vec{x}, t) &= +[\partial_\mu \pi^\mu(\vec{x}, t) + \frac{q}{i\hbar} \pi_\psi(\vec{x}, t) \psi(\vec{x}, t)]
\end{aligned}$$

We can choose the positive sign since at the end of the calculations the constraint is forced to be zero.

3- The third constraint is the gauge condition.

The gauge condition constrains the dynamics by imposing some restricting conditions on the scalar and vector potentials. We work in the Poincaré gauge where the potential vector is orthogonal to any of the observation points: $\vec{x}.\vec{A}(\vec{x}, t) = 0$. So the third constraint reads:

$$\chi_3(\vec{x}, t) = x_\mu A^\mu(\vec{x}, t)$$

4- The fourth and last constraint derives from the previous one since $\chi_3(\vec{x}, t)$ has to remain true at any time:

$$\begin{aligned}
\chi_4(\vec{x}, t) &= \{\chi_3, \mathcal{H}\}(\vec{x}, t) = \frac{\chi_3}{\delta A^\mu} \frac{\delta \mathcal{H}}{\delta \pi^\mu} \\
&= \frac{1}{\varepsilon_0} x_\mu \pi^\mu(\vec{x}, t) - x_\mu \partial^\mu \phi(\vec{x}, t)
\end{aligned}$$

C. The matrix of constraints C and its inverse K

In order to compute the time-evolution of any function in the phase-space restricted by the constraints, a redefinition of the Poisson brackets is needed. This is done by computing the Dirac brackets. The first step for this computation is to define the matrix of constraint **C** that takes into account for all the constraints. We also need to define the inverse **K** of the matrix of constraints **C**.

The coefficients of the matrix of constraints **C** are defined through the Poisson brackets evaluated for two different constraints as $C_{i,j}(\vec{x},\vec{y},t) = \{\chi_i(\vec{x},t), \chi_j(\vec{y},t)\}$ see p.314 in [14].

We need to define the Poisson brackets evaluated at two different points $\vec{x}$ and $\vec{y}$:

$$\begin{aligned}
\{\chi_1(\vec{x},t), \chi_2(\vec{y},t)\} &= \int d\vec{u} \frac{\delta\chi_1[\psi(\vec{x}),\pi_\psi(\vec{x})]}{\delta\psi(\vec{u})} \frac{\delta\chi_2[\psi(\vec{y}),\pi_\psi(\vec{y})]}{\delta\pi_\psi(\vec{u})} - \frac{\delta\chi_2[\psi(\vec{y}),\pi_\psi(\vec{y}))]}{\delta\psi(\vec{u})} \frac{\delta\chi_1[\psi(\vec{x}),\pi_\psi(\vec{x})]}{\delta\pi_\psi(\vec{u})} \\
&+ (\psi \to \phi) \ ; \ (\pi_\psi \to \pi_\phi) \\
&+ (\psi \to A^\mu) \ ; \ (\pi_\psi \to \pi_\mu)
\end{aligned}$$

The functionals χ_1 and χ_2 are defined on the phase-space and depend on all fields (including their gradients) and their conjugated momenta (including their gradients).

The non-zero elements of the matrix of constraints **C** are:

$$\begin{aligned}
C_{1,4}(\vec{x},\vec{z},t) &= \{\chi_1(\vec{x},t), \chi_4(\vec{z},t)\} = \int d\vec{u}\ \delta(\vec{x}-\vec{u}) z_\mu \partial^\mu_{\vec{z}}[\delta(\vec{z}-\vec{u})] = z_\mu \partial^\mu_{\vec{z}}[\delta(\vec{z}-\vec{x})] \\
C_{2,3}(\vec{x},\vec{z},t) &= \{\chi_2(\vec{x},t), \chi_3(\vec{z},t)\} = -\int d\vec{u}\ \partial^{\vec{x}}_\mu[\delta(\vec{x}-\vec{u})] z^\mu \delta(\vec{z}-\vec{u}) = -z^\mu \partial^{\vec{x}}_\mu[\delta(\vec{x}-\vec{z})] \\
C_{3,4}(\vec{x},\vec{z},t) &= \{\chi_3(\vec{x},t), \chi_4(\vec{z},t)\} = \frac{1}{\varepsilon_0} \int d\vec{u}\ x_\mu \delta(\vec{x}-\vec{u}) z^\mu \delta(\vec{z}-\vec{u}) = \frac{1}{\varepsilon_0} x_\mu z^\mu \delta(\vec{x}-\vec{z})
\end{aligned}$$

The inverse of the matrix of constraints **K** is defined in the following way (see p.314 in ref.[14]):

$$\int d\vec{z} \mathbf{C}(\vec{x},\vec{z},t).\mathbf{K}(\vec{z},\vec{y},t) = \delta(\vec{x}-\vec{y}) \mathbf{I}_4$$

where $\delta(\vec{x}-\vec{y})$ is the Dirac distribution and $\mathbf{I}_4$ is the 4×4 identity-matrix.

The matrix **K** reads:

$$\mathbf{K}(\vec{z},\vec{y},t) = \begin{pmatrix} 0 & K_{1,2}(\vec{z},\vec{y},t) & 0 & K_{1,4}(\vec{z},\vec{y},t) \\ K_{2,1}(\vec{z},\vec{y},t) & 0 & K_{2,3}(\vec{z},\vec{y},t) & 0 \\ 0 & K_{3,2}(\vec{z},\vec{y},t) & 0 & 0 \\ K_{4,1}(\vec{z},\vec{y},t) & 0 & 0 & 0 \end{pmatrix}$$

Its product with the matrix of constraints **C** leads to:

$$\int d\vec{z} \mathbf{C}(\vec{x},\vec{z},t).\mathbf{K}(\vec{z},\vec{y},t) = \begin{pmatrix} -\partial_\mu[x^\mu K_{4,1}] & 0 & 0 & 0 \\ 0 & -\partial_\mu[x^\mu K_{3,2}] & 0 & 0 \\ -\partial_\mu[x^\mu K_{2,1}] + \frac{1}{\varepsilon_0} x_\mu x^\mu K_{4,1} & 0 & \partial_\mu[x^\mu K_{2,3}] & 0 \\ 0 & \partial_\mu[x^\mu K_{1,2}] - \frac{1}{\varepsilon_0} x_\mu x^\mu K_{2,3} & 0 & \partial_\mu[x^\mu K_{1,4}] \end{pmatrix}$$

The diagonal terms comes from $\int d\vec{z} C_{i,5-i}(\vec{x},\vec{z}) K_{5-i,i}(\vec{z},\vec{y}) = \delta(\vec{x}-\vec{y})$ with $i = 1...4$, whereas the non-diagonal terms come from $\int d\vec{z}[C_{3,2}(\vec{x},\vec{z}) K_{2,1}(\vec{z},\vec{y}) + C_{3,4} K_{4,1}(\vec{z},\vec{y})] = 0$ and $\int d\vec{z}[C_{4,1}(\vec{x},\vec{z}) K_{1,2}(\vec{z},\vec{y}) + C_{4,3} K_{3,2}(\vec{z},\vec{y})] = 0$.

Because the matrix of constraints **C** and its inverse are anti-symmetric, the following properties hold:

$$K_{4,1}(\vec{x},\vec{y}) = -K_{1,4}(\vec{x},\vec{y}) = K_{3,2}(\vec{x},\vec{y}) = -K_{2,3}(\vec{x},\vec{y})$$

and

$$K_{1,2}(\vec{x},\vec{y}) = -K_{2,1}(\vec{x},\vec{y})$$

Finally, we have to solve only two equations:

$$\partial_\mu^{\vec{x}}[x^\mu K_{2,3}(\vec{x},\vec{y})] = \delta(\vec{x}-\vec{y}) \tag{19}$$
$$\partial_\mu^{\vec{x}}[x^\mu K_{1,2}(\vec{x},\vec{y})] = \frac{1}{\varepsilon_0} x_\mu x^\mu K_{3,2}(\vec{x},\vec{y}) \tag{20}$$

The previous relations eq:(19) and eq:(20) are actually enough to compute the dynamical equations from the commutators and the Heisenberg equation. We could stop here. Indeed, no explicit solution of these equations is needed to compute the dynamical equations since the calculations only involve relationships between the elements of the matrix **K**. See the paragraph *" IIIF: Dynamical equations"* for illustrations.

Nevertheless, a solution to the equation eq:(19) can be found.

First we solve eq:(19) in spherical coordinates and finally express the solution in cartesian coordinates. Two points namely X and Y are involved in this calculation. The point X is specified by its distance from the origin $\vec{x} = x\vec{e}_r$ and the angles θ_x, φ_x whereas the point Y is specified by $\vec{y} = y\vec{e}_r$ and the angles θ_y, φ_y.

The equation eq:(19) actually involves the divergence operator:

$$\vec{\nabla}_{\vec{x}}.[\vec{x}K_{2,3}(\vec{x},\vec{y})] = \delta(\vec{x}-\vec{y})$$

that reads in spherical coordinates:

$$\frac{1}{x^2\sin\theta_x}\partial_x[x^2\sin(\theta_x)xK_{2,3}(\vec{x},\vec{y})] = \frac{1}{x^2\sin\theta_x}\delta(x-y)\delta(\theta_x-\theta_y)\delta(\varphi_x-\varphi_y)$$

The Dirac distribution is also written in spherical coordinates. This equation can be integrated. For simplicity we assume no additional constant or function that could depend only on angular variables θ_x, φ_x :

$$x^2\sin(\theta_x)xK_{2,3}(\vec{x},\vec{y}) = H(x-y)\delta(\theta_x-\theta_y)\delta(\varphi_x-\varphi_y)$$

Where $H(x-y)$ is the Heaviside distribution.

$$K_{2,3}(\vec{x},\vec{y}) = \frac{1}{x^2\sin(\theta_x)}\frac{1}{x}H(x-y)\delta(\theta_x-\theta_y)\delta(\varphi_x-\varphi_y)$$

In cartesian units, points X and Y are specified respectively by a triplet of three numbers $X = (x_1, x_2, x_3)$ and $Y = (y_1, y_2, y_3)$.

Knowing that $\theta_x = \arccos(\frac{x_3}{|\vec{x}|})$ and $\varphi_x = \arctan(\frac{x_2}{|\vec{x}|})$, and remarking that the Jacobian is $x^2\sin(\theta_x)$ the solution in cartesian coordinates can be written as:

$$K_{2,3}(\vec{x},\vec{y}) = \frac{1}{|\vec{x}|}H(|\vec{x}|-|\vec{y}|)\delta(x_2-y_2)\delta(x_3-y_3)$$

D. The Dirac brackets

We denote $\{.,.\}_D$ the Dirac brackets. They derived from the Poisson brackets with the following definition[14, 20] :

$$\{A^\mu(\vec{x},t),\pi_\nu(\vec{y},t)\}_D = \{A^\mu(\vec{x},t),\pi_\nu(\vec{y},t)\} - \sum_{i,j=1}^{4}\{A^\mu,\chi_i\}(\vec{x},t)K_{i,j}(\vec{x},\vec{y})\{\chi_j,\pi_\nu\}(\vec{y},t)$$

We now compute de Dirac brackets between the different dynamical variables.

a. Dirac brackets between the potential vector $A^\mu(\vec{x},t)$ and its conjugated momentum $\pi_\nu(\vec{y},t)$.
The only non-vanishing Poisson brackets are the one between $A^\mu(\vec{x},t)$ and $\chi_2(\vec{x},t)$ and the one between $\pi_\nu(\vec{y},t)$ and $\chi_3(\vec{y},t)$

$$\{A^\mu, \chi_2\}(\vec{x},t) = \frac{\delta A^\mu}{\delta A^\mu}\frac{\delta \chi_2}{\delta \pi_\mu} = -\partial^\mu_{\vec{x}}[.]$$

$$\{\chi_3, \pi_\nu\}(\vec{y},t) = \frac{\delta \chi_3}{\delta A^\nu}\frac{\delta \pi_\nu}{\delta \pi_\nu} = y_\nu$$

The Dirac bracket is then:

$$\begin{aligned}
\{A^\mu(\vec{x},t), \pi_\nu(\vec{y},t)\}_D &= \{A^\mu(\vec{x},t), \pi_\nu(\vec{y},t)\} - \{A^\mu, \chi_2\}(\vec{x},t) K_{23}(\vec{x},\vec{y})\{\chi_3, \pi_\nu\}(\vec{y},t) \\
&= \delta^\mu_\nu \delta(\vec{x}-\vec{y}) + \partial^\mu_{\vec{x}}[y_\nu K_{23}(\vec{x},\vec{y})] \\
\{A^\mu(\vec{x},t), \pi_\nu(\vec{y},t)\}_D &= \delta^\mu_\nu \delta(\vec{x}-\vec{y}) + y_\nu \partial^\mu_{\vec{x}}[K_{23}(\vec{x},\vec{y})]
\end{aligned}$$

b. Dirac brackets between $\psi(\vec{x},t)$ and $\pi^\mu(\vec{y},t)$.
The only non-vanishing Poisson brackets between the dynamical variables and the constraints are $\{\psi, \chi_2\}(\vec{x},t)$ and $\{\chi_3, \pi^\mu\}(\vec{y},t)$. The Dirac brackets between $\psi(\vec{x},t)$ and $\pi^\mu(\vec{y},t)$ is finally:

$$\{\psi(\vec{x},t), \pi^\mu(\vec{y},t)\}_D = \{\psi(\vec{x},t), \pi^\mu(\vec{y},t)\} - \{\psi, \chi_2\}(\vec{x},t) K_{2,3}(\vec{x},\vec{y})\{\chi_3, \pi^\mu\}(\vec{y},t)$$

$$\{\psi, \chi_2\}(\vec{x},t) = \frac{\delta \psi}{\delta \psi}\frac{\delta \chi_2}{\delta \pi_\psi} = \frac{q}{i\hbar}\psi(\vec{x},t)$$

Now we compute the other Poisson bracket:

$$\{\chi_3, \pi^\mu\}(\vec{y},t) = \frac{\delta \chi_3}{\delta A_\mu}\frac{\delta \pi^\mu}{\delta \pi^\mu} = y^\mu$$

So the Dirac bracket is given by:

$$\{\psi(\vec{x},t), \pi^\mu(\vec{y},t)\}_D = -\frac{q}{i\hbar} y^\mu K_{2,3}(\vec{x},\vec{y})\psi(\vec{x},t)$$

In the case of the field conjugated to the Schrödinger field, $\pi_\psi(\vec{x},t)$, a similar calculation leads to:

$$\{\pi_\psi(\vec{x},t), \pi^\mu(\vec{y},t)\}_D = \frac{q}{i\hbar} y^\mu K_{2,3}(\vec{x},\vec{y})\pi_\psi(\vec{x},t)$$

Note that in this gauge, the electromagnetic field degrees of freedom don't commute with the matter degrees of freedom. There is a similar result in the Coulomb gauge if one quantizes the total conjugated momentum (transverse+longitudinal part) π^μ. The coupling between field and matter degrees of freedom can be removed in the Coulomb gauge by quantifying only the transverse part of the conjugated momentum $\pi^{\mu\perp}$ (see Weinberg[14] p.316). In the Poincaré gauge, the Dirac brackets are non-vanishing because π^μ and ψ are not independent variables. They are link through de Maxwell-Gauss equation (constraint χ_2).

E. Commutation relations between quantum operators

The canonical quantization procedure to produce a quantum theory from a classical theory consists in assuming that the commutator denoted $[\bullet,\bullet]$ between two operators is given by the results of the Dirac brackets times $i\hbar$. The particles being fermions, we impose anti-commutator, denoted $[\bullet,\bullet]_+$, between the Schrödinger field operator $\hat{\psi}(\vec{x},t)$ and its conjugated momentum $\hat{\pi}_\psi(\vec{x},t)$.

Finally, the commutation and anti-commutation rules in the Poincaré gauge are given by:

$$\begin{aligned}
[\hat{A}^\mu(\vec{x},t),\hat{\pi}_\nu(\vec{y},t)] &= i\hbar[\delta^\mu_\nu\delta(\vec{x}-\vec{y}) + y_\nu\partial^\mu_{\vec{x}}K_{2,3}(\vec{x},\vec{y})] \\
[\hat{\psi}(\vec{x},t),\hat{\pi}^\mu(\vec{y},t)] &= -qy^\mu K_{2,3}(\vec{x},\vec{y})\hat{\psi}(\vec{x},t) \\
[\hat{\pi}_\psi(\vec{x},t),\hat{\pi}^\mu(\vec{y},t)] &= qy^\mu K_{2,3}(\vec{x},\vec{y})\hat{\pi}_\psi(\vec{x},t) \\
[\hat{\psi}(\vec{x},t),\hat{\pi}_\psi(\vec{y},t)]_+ &= i\hbar\delta(\vec{x}-\vec{y})
\end{aligned}$$

The commutators between the physical fields can be deduced from the previous results. As an example, we derived the commutator between $\hat{B}_2(\vec{x},t)$ and $\hat{\pi}^1(\vec{y},t)$:

$$\begin{aligned}
[\hat{B}_2(\vec{x},t),\hat{\pi}^1(\vec{y},t)] &= [\partial^{\vec{x}}_3\hat{A}_1(\vec{x},t) - \partial^{\vec{x}}_1\hat{A}_3(\vec{x},t),\hat{\pi}^1(\vec{y},t)] \\
&= \partial^{\vec{x}}_3[\hat{A}_1(\vec{x},t),\hat{\pi}^1(\vec{y},t)] - \partial^{\vec{x}}_1[\hat{A}_3(\vec{x},t),\hat{\pi}^1(\vec{y},t)] \\
&= i\hbar\partial^{\vec{x}}_3[\delta(\vec{x}-\vec{y}) + y_1\partial^1_{\vec{x}}K_{23}(\vec{x},\vec{y})] - i\hbar\partial^{\vec{x}}_1[y_1\partial^3_{\vec{x}}K_{23}(\vec{x},\vec{y})] \\
&= i\hbar\partial^{\vec{x}}_3\delta(\vec{x}-\vec{y}) + i\hbar(y_1\partial^{\vec{x}}_3\partial^1_{\vec{x}}[K_{23}(\vec{x},\vec{y})] - y_1\partial^3_{\vec{x}}\partial^{\vec{x}}_1[K_{23}(\vec{x},\vec{y})]) \\
[\hat{\pi}^1(\vec{y},t),\hat{B}_2(\vec{x},t)] &= -i\hbar\partial^{\vec{x}}_3[\delta(\vec{x}-\vec{y})] \\
[\hat{E}^1(\vec{y},t),\hat{B}_2(\vec{x},t)] &= \frac{i\hbar}{\varepsilon_0}\partial^{\vec{x}}_3[\delta(\vec{x}-\vec{y})] = \frac{i\hbar}{\varepsilon_0}\frac{\partial}{\partial x_3}[\delta(\vec{x}-\vec{y})] \\
[\hat{E}^1(\vec{y},t),\hat{B}_2(\vec{x},t)] &= -\frac{i\hbar}{\varepsilon_0}\partial^{\vec{y}}_3[\delta(\vec{x}-\vec{y})] = -\frac{i\hbar}{\varepsilon_0}\frac{\partial}{\partial y_3}[\delta(\vec{x}-\vec{y})]
\end{aligned} \tag{21}$$

Cyclic permutations occurs.

This is exactly the same result as in the Coulomb gauge [2, 14]. As expected, the commutation relations between the physical fields are independent of the gauge conditions.

F. Dynamical equations

The dynamical equations for the quantum operators are derived from the Heisenberg equations with the help of the hamiltonian density operator $\hat{\mathcal{H}}(\vec{y},t)$. Since the commutators have been computed through the Dirac brackets, they take all constraints into account. Then, the hamiltonian density operator can be simplify with the help of the Maxwell-gauss equation (*i.e.* constraint χ_2). It finally reads:

$$\hat{\mathcal{H}}(\vec{y},t) = \frac{\hat{\pi}^\dagger_\psi(\vec{y},t)}{i\hbar}\{-\frac{\hbar^2}{2m}[\partial^{\vec{y}}_\mu - \frac{iq}{\hbar}\hat{A}_\mu(\vec{y},t)]^2 + V(\vec{y},t)\}\hat{\psi}(\vec{y},t) + \frac{1}{2\varepsilon_0}\hat{\pi}^\mu(\vec{y},t)\hat{\pi}_\mu(\vec{y},t) + \frac{1}{2\mu_0}\hat{B}\mu(\vec{y},t)\hat{B}_\mu(\vec{y},t) \tag{22}$$

To simplify the writing, we define the differential operator $\mathcal{F}(\bullet) = -\frac{\hbar^2}{2m}[\partial^{\vec{y}}_\mu - \frac{iq}{\hbar}\hat{A}_\mu(\vec{y},t)]^2 + V(\vec{y},t)$ that applies to the Schrödinger operator $\hat{\psi}(\vec{y},t)$. The hamiltonian density reads:

$$\mathcal{H}(\vec{y},t) = \frac{\hat{\pi}^\dagger_\psi(\vec{y},t)}{i\hbar}\mathcal{F}(\hat{\psi}(\vec{y},t)) + \frac{1}{2\varepsilon_0}\hat{\pi}^2(\vec{y},t) + \frac{1}{2\mu_0}\hat{B}^2(\vec{y},t)$$

1. The vector potential

We derive here the dynamical equation satisfied by the vector potential.

$$\begin{aligned}
i\hbar\dot{\hat{A}}^\mu(\vec{x},t) &= \int d\vec{y}[\hat{A}^\mu(\vec{x},t),\hat{\mathcal{H}}(\vec{y})] \\
&= \int d\vec{y}[\hat{A}^\mu(\vec{x},t),\hat{\pi}_\nu(\vec{y},t)]\frac{\hat{\pi}^\nu(\vec{y},t)}{\varepsilon_0} \\
&= i\hbar\int d\vec{y}\big(\delta^\mu_\nu\delta(\vec{x}-\vec{y})+y_\nu\partial^\mu_{\vec{x}}[K_{23}(\vec{x},\vec{y})]\big)\frac{\hat{\pi}^\nu(\vec{y},t)}{\varepsilon_0} \\
&= i\hbar\frac{\pi^\mu(\vec{x},t)}{\varepsilon_0}+i\hbar\partial^\mu_{\vec{x}}\Big[\int d\vec{y}K_{23}(\vec{x},\vec{y})y_\nu\frac{\hat{\pi}^\nu(\vec{y},t)}{\varepsilon_0}\Big]
\end{aligned} \tag{23}$$

To arrive at this equation we have used the following identity: $[A,BC]=B[A,C]+[A,B]C$.
From the constraint χ_4, one has $\frac{1}{\varepsilon_0}y_\nu\hat{\pi}^\nu(\vec{y},t)=y_\nu\partial^\nu_{\vec{y}}\hat{\phi}(\vec{y},t)$. So the calculation leads to:

$$\begin{aligned}
\dot{\hat{A}}^\mu(\vec{x},t) &= \frac{\hat{\pi}^\mu(\vec{x},t)}{\varepsilon_0}+\partial^\mu_{\vec{x}}\Big[\int d\vec{y}K_{23}(\vec{x},\vec{y})y_\nu\partial^\nu_{\vec{y}}\hat{\phi}(\vec{y},t)\Big] \\
&= \frac{\hat{\pi}^\mu(\vec{x},t)}{\varepsilon_0}-\partial^\mu_{\vec{x}}\Big[\int d\vec{y}\partial^\nu_{\vec{y}}[y_\nu K_{23}(\vec{x},\vec{y})]\hat{\phi}(\vec{y},t)\Big]
\end{aligned} \tag{24}$$

From the calculations of the Dirac brackets, the coefficient K_{23} satisfies $\partial^\nu_{\vec{y}}[y_\nu K_{23}(\vec{x},\vec{y})]=\vec{\nabla}_{\vec{y}}.[\vec{y}K_{23}(\vec{x},\vec{y})]=+\delta(\vec{y}-\vec{x})$.
The dynamical equation satisfied by the vector potential is finally given by:

$$\dot{\hat{A}}^\mu(\vec{x},t) = \frac{\hat{\pi}^\mu(\vec{x},t)}{\varepsilon_0}-\partial^\mu_{\vec{x}}\hat{\phi}(\vec{y},t)$$

which is similar to the equation satisfied by the classical vector-potential. As noticed previously, only equation (19) was needed to compute the dynamical equation and not the explicit expression of $K_{2,3}(\vec{x},\vec{y})$.

2. The Schrödinger equation

With the help of the anti-commutation relation between $\hat{\psi}$ and $\hat{\pi}_\psi$, *i.e.* $[\hat{\psi}(\vec{x},t),\hat{\pi}_\psi(\vec{y},t)]_+=i\hbar\delta(\vec{x}-\vec{y})$, and the commutation relation between ψ and the electric field degrees of freedom $\hat{\pi}^\mu(\vec{y},t)$, $[\hat{\psi}(\vec{x},t),\hat{\pi}^\mu(\vec{y},t)]=-qy\mu K_{2,3}(\vec{x},\vec{y})\hat{\psi}(\vec{x},t)$, the Schrödinger equation satisfied by the Schrödinger-field operator $\hat{\psi}(\vec{x},t)$ can be found. Indeed,

$$\begin{aligned}
i\hbar\dot{\hat{\psi}}(\vec{x},t) &= \int d\vec{y}[\hat{\psi}(\vec{x},t),\hat{\mathcal{H}}(\vec{y},t)] \\
&= \int d\vec{y}[\hat{\psi}(\vec{x},t),\frac{\hat{\pi}^\dagger_\psi(\vec{y},t)}{i\hbar}\mathcal{F}(\hat{\psi}(\vec{y},t))+\frac{1}{2\varepsilon_0}\hat{\pi}^2_\mu(\vec{y},t)] \\
&= \int d\vec{y}[\hat{\psi}(\vec{x},t),\frac{\hat{\pi}^\dagger_\psi(\vec{y},t)}{i\hbar}]_+\ \mathcal{F}(\hat{\psi}(\vec{y},t))-\frac{\hat{\pi}^\dagger_\psi(\vec{y},t)}{i\hbar}[\hat{\psi}(\vec{x},t),\mathcal{F}(\hat{\psi}(\vec{y},t))]_+ + \\
&\quad \frac{1}{\varepsilon_0}[\hat{\psi}(\vec{x},t),\hat{\pi}^\mu(\vec{y},t)]\hat{\pi}_\mu(\vec{y},t)
\end{aligned}$$

Here we have used the following relationship between commutators and anticommutators: $[A,BC]=B[A,C]_+-[A,B]_+C$.
We notice that $[\hat{\psi}(\vec{x},t),\mathcal{F}(\hat{\psi}(\vec{y},t))]_+=\mathcal{F}([\hat{\psi}(\vec{x},t),\hat{\psi}(\vec{y},t)]_+)=0$ since $[\hat{\psi}(\vec{x},t),\hat{\psi}(\vec{y},t)]_+=0$. As a consequence, recalling that $[\hat{\psi}(\vec{x},t),\hat{\pi}_\psi(\vec{y},t)]_+=i\hbar\delta(\vec{x}-\vec{y})$ the previous calculation simplifies as:

$$
\begin{aligned}
i\hbar\dot{\hat{\psi}}(\vec{x},t) &= \big[-\frac{\hbar^2}{2m}(\partial_\mu^{\vec{x}} - \frac{iq}{\hbar}\hat{A}_\mu(\vec{x},t))^2 + V(\vec{x})\big]\hat{\psi}(\vec{x},t) + \int d\vec{y}[\hat{\psi}(\vec{x},t),\hat{\pi}^\mu(\vec{y},t)]\frac{\hat{\pi}_\mu(\vec{y},t)}{\varepsilon_0} \\
&= \big[-\frac{\hbar^2}{2m}(\partial_\mu^{\vec{x}} - \frac{iq}{\hbar}\hat{A}_\mu(\vec{x},t))^2 + V(\vec{x})\big]\hat{\psi}(\vec{x},t) - q\hat{\psi}(\vec{x},t)\int d\vec{y}y^\mu K_{23}(\vec{x},\vec{y})\frac{\hat{\pi}_\mu(\vec{y},t)}{\varepsilon_0}
\end{aligned}
$$

Again with the help of $\chi_4(\vec{y},t)$, $\frac{1}{\varepsilon_0}y^\mu\hat{\pi}_\mu(\vec{y},t) = y^\mu\partial_\mu\vec{y}\hat{\phi}(\vec{y},t)$

$$
\begin{aligned}
i\hbar\dot{\hat{\psi}}(\vec{x},t) &= \big[-\frac{\hbar^2}{2m}(\partial_\mu^{\vec{x}} - \frac{iq}{\hbar}\hat{A}_\mu(\vec{x},t))^2 + V(\vec{x})\big]\hat{\psi}(\vec{x},t) - q\hat{\psi}(\vec{x},t)\int d\vec{y}K_{23}(\vec{x},\vec{y})y_\mu\partial_{\vec{y}}^\mu\hat{\phi}(\vec{y},t) \\
&= \big[-\frac{\hbar^2}{2m}(\partial_\mu^{\vec{x}} - \frac{iq}{\hbar}\hat{A}_\mu(\vec{x},t))^2 + V(\vec{x})\big]\hat{\psi}(\vec{x},t) + q\hat{\psi}(\vec{x},t)\int d\vec{y}\partial_{\vec{y}}^\mu[y_\mu K_{23}(\vec{x},\vec{y})]\hat{\phi}(\vec{y},t)
\end{aligned}
$$

The calculation of the Dirac brackets leads to the result : $\partial_{\vec{y}}^\mu[y_\mu K_{23}(\vec{x},\vec{y})] = \delta(\vec{y}-\vec{x})$ which ables to recover the Schrödinger equation satisfied by the Schrödinger-field operator.

$$
i\hbar\dot{\hat{\psi}}(\vec{x},t) = \big[-\frac{\hbar^2}{2m}(\partial_\mu^{\vec{x}} - \frac{iq}{\hbar}\hat{A}_\mu(\vec{x},t))^2 + V(\vec{x})\big]\hat{\psi}(\vec{x},t) + q\hat{\phi}(\vec{x},t)\hat{\psi}(\vec{x},t)
$$

3. The Maxwell-Ampère equation

The derivation is made easier if we integrate by parts the kinetic energy term of the equation:(22). Using the definition of the canonical conjugate momentum $\hat{\pi}_\psi(\vec{y},t) = i\hbar\hat{\psi}^\dagger(\vec{y},t)$, the hamiltonian density reads:

$$
\begin{aligned}
\hat{\mathcal{H}}(\vec{y},t) &= \frac{\hbar^2}{2m}[\partial_\mu^{\vec{y}} + \frac{iq}{\hbar}\hat{A}_\mu(\vec{y},t)]\hat{\psi}^\dagger(\vec{y},t) \times [\partial_\mu^{\vec{y}} - \frac{iq}{\hbar}\hat{A}_\mu(\vec{y},t)]\hat{\psi}(\vec{y},t) \\
&+ \hat{\psi}^\dagger(\vec{y},t)V(\vec{y},t)\hat{\psi}(\vec{y},t) \\
&+ \frac{1}{2\varepsilon_0}\hat{\pi}^\mu(\vec{y},t)\hat{\pi}_\mu(\vec{y},t) + \frac{1}{2\mu_0}\hat{B}\mu(\vec{y},t)\hat{B}_\mu(\vec{y},t)
\end{aligned}
$$

We introduce the following notation $D_\mu[\hat{\psi}] = \partial_\mu^{\vec{y}}\hat{\psi}(\vec{y},t) - \frac{iq}{\hbar}\hat{A}_\mu(\vec{y},t)\hat{\psi}(\vec{y},t)$ and $D_\mu^\star[\hat{\psi}^\dagger] = \partial_\mu^{\vec{y}}\hat{\psi}^\dagger(\vec{y},t) + \frac{iq}{\hbar}\hat{A}_\mu(\vec{y},t)\hat{\psi}^\dagger(\vec{y},t)$

The calculation needs the following commutators:

$$
\begin{aligned}
[\hat{\pi}^\nu(\vec{x},t),\hat{\psi}^\dagger(\vec{y},t)] &= -qx^\nu K_{2,3}(\vec{x},\vec{y})\psi^\dagger(\vec{y},t) \\
[\hat{\pi}^\nu(\vec{x},t),\hat{\psi}(\vec{y},t)] &= qx^\nu K_{2,3}(\vec{x},\vec{y})\psi(\vec{y},t) \\
[\hat{\pi}^\nu(\vec{x},t),\hat{A}_\mu(\vec{y},t)] &= -i\hbar\{\delta_\mu^\nu\delta(\vec{x}-\vec{y}) + x^\nu\partial_\mu^{\vec{y}}K_{2,3}(\vec{x},\vec{y})\}
\end{aligned}
$$

First we compute the commutator between the canonical momentum $\hat{\pi}^\nu(\vec{x},t)$ and the conjugate of covariant derivative $D_\mu^\star[\hat{\psi}^\dagger]$. One finds:

$$
\begin{aligned}
[\hat{\pi}^\nu(\vec{x},t),D_\mu^\star[\hat{\psi}^\dagger]] &= \partial_\mu^{\vec{y}}[\hat{\pi}^\nu(\vec{x},t),\hat{\psi}^\dagger(\vec{y},t)] + \frac{iq}{\hbar}[\hat{\pi}^\nu(\vec{x},t),\hat{A}_\mu(\vec{y},t)]\hat{\psi}^\dagger(\vec{y},t) + \frac{iq}{\hbar}\hat{A}_\mu(\vec{y},t)[\hat{\pi}^\nu(\vec{x},t),\hat{\psi}^\dagger(\vec{y},t)] \\
&= -qx^\nu\partial_\mu^{\vec{y}}[K_{2,3}(\vec{x},\vec{y})\psi^\dagger(\vec{y},t)] + q\{\delta_\mu^\nu\delta(\vec{x}-\vec{y}) + x^\nu\partial_\mu^{\vec{y}}K_{2,3}(\vec{x},\vec{y})\}\hat{\psi}^\dagger(\vec{y},t) - q\frac{iq}{\hbar}\hat{A}_\mu(\vec{y},t)x^\nu K_{2,3}(\vec{x},\vec{y})\psi^\dagger(\vec{y},t) \\
&= -qx^\nu K_{2,3}(\vec{x},\vec{y})\partial_\mu^{\vec{y}}[\psi^\dagger(\vec{y},t)] + q\delta_\mu^\nu\delta(\vec{x}-\vec{y})\hat{\psi}^\dagger(\vec{y},t) - q\frac{iq}{\hbar}\hat{A}_\mu(\vec{y},t)x^\nu K_{2,3}(\vec{x},\vec{y})\psi^\dagger(\vec{y},t) \\
&= -qx^\nu K_{2,3}(\vec{x},\vec{y})\{\partial_\mu^{\vec{y}}[\psi^\dagger(\vec{y},t)] + \frac{iq}{\hbar}\hat{A}_\mu(\vec{y},t)\psi^\dagger(\vec{y},t)\} + q\delta_\mu^\nu\delta(\vec{x}-\vec{y})\hat{\psi}^\dagger(\vec{y},t) \\
&= -qx^\nu K_{2,3}(\vec{x},\vec{y})D_\mu^\star[\psi^\dagger] + q\delta_\mu^\nu\delta(\vec{x}-\vec{y})\hat{\psi}^\dagger(\vec{y},t)
\end{aligned}
$$

Now we compute the commutator between the canonical conjugate momentum $\hat{\pi}^\nu(\vec{x},t)$ and the covariant derivative $D_\mu[\hat{\psi}]$. One finds:

$$\begin{aligned}
[\hat{\pi}^\nu(\vec{x},t), D_\mu[\hat{\psi}]] &= \partial_\mu^{\vec{y}}[\hat{\pi}^\nu(\vec{x},t), \hat{\psi}(\vec{y},t)] - \frac{iq}{\hbar}[\hat{\pi}^\nu(\vec{x},t), \hat{A}(\vec{y},t)]\hat{\psi}(\vec{y},t) - \frac{iq}{\hbar}\hat{A}_\mu(\vec{y},t)[\hat{\pi}^\nu(\vec{x},t), \hat{\psi}(\vec{y},t)] \\
&= qx^\nu\partial_\mu^{\vec{y}}[K_{2,3}(\vec{x},\vec{y})\psi(\vec{y},t)] - q\{\delta_\mu^\nu\delta(\vec{x}-\vec{y}) + x^\nu\partial_\mu^{\vec{y}}K_{2,3}(\vec{x},\vec{y})\}\hat{\psi}(\vec{y},t) - q\frac{iq}{\hbar}\hat{A}_\mu(\vec{y},t)x^\nu K_{2,3}(\vec{x},\vec{y})\psi(\vec{y},t) \\
&= qx^\nu K_{2,3}(\vec{x},\vec{y})\partial_\mu^{\vec{y}}[\psi(\vec{y},t)] - q\delta_\mu^\nu\delta(\vec{x}-\vec{y})\hat{\psi}(\vec{y},t) - q\frac{iq}{\hbar}\hat{A}_\mu(\vec{y},t)x^\nu K_{2,3}(\vec{x},\vec{y})\psi(\vec{y},t) \\
&= qx^\nu K_{2,3}(\vec{x},\vec{y})\{\partial_\mu^{\vec{y}}[\psi(\vec{y},t)] - \frac{iq}{\hbar}\hat{A}_\mu(\vec{y},t)\psi(\vec{y},t)\} - q\delta_\mu^\nu\delta(\vec{x}-\vec{y})\hat{\psi}(\vec{y},t) \\
&= qx^\nu K_{2,3}(\vec{x},\vec{y})D_\mu[\psi] - q\delta_\mu^\nu\delta(\vec{x}-\vec{y})\hat{\psi}(\vec{y},t)
\end{aligned}$$

The Heisenberg equation, with the help of the hamiltonian density $\hat{\mathcal{H}}(\vec{y},t)$ will involve the two following commutators:

$$\left[\hat{\pi}^\nu(\vec{x},t), D_\mu^\star[\hat{\psi}^\dagger]\right] D_\mu[\hat{\psi}] = -qx^\nu K_{2,3}(\vec{x},\vec{y})D_\mu^\star[\psi^\dagger]D_\mu[\hat{\psi}] + q\delta_\mu^\nu\delta(\vec{x}-\vec{y})\hat{\psi}^\dagger(\vec{y},t)D_\mu[\hat{\psi}]$$

And,

$$D_\mu^\star[\hat{\psi}^\dagger]\left[\hat{\pi}^\nu(\vec{x},t), D_\mu[\hat{\psi}]\right] = qx^\nu K_{2,3}(\vec{x},\vec{y})D_\mu^\star[\hat{\psi}^\dagger]D_\mu[\psi] - q\delta_\mu^\nu\delta(\vec{x}-\vec{y})D_\mu^\star[\hat{\psi}^\dagger]\hat{\psi}(\vec{y},t)$$

Finally, with the help of the previous results, the time-evolution equation of the canonical conjugate momentum $\pi^\nu(\vec{x},t)$ leads to the Maxwell-Ampère equation:

$$\begin{aligned}
i\hbar\frac{d}{dt}\hat{\pi}^\nu(\vec{x},t) &= \frac{\hbar^2}{2m}q\{\hat{\psi}^\dagger(\vec{x},t)D^\nu[\hat{\psi}] - D^{\star,\nu}[\hat{\psi}^\dagger]\hat{\psi}(\vec{x},t)\} - \frac{i\hbar}{\mu_0}\vec{\nabla}\times\hat{B}(\vec{x},t)|^\nu \\
-\varepsilon_0\frac{d}{dt}\hat{E}^\nu(\vec{x},t) &= -i\frac{\hbar}{2m}q\{\hat{\psi}^\dagger(\vec{x},t)D^\nu[\hat{\psi}] - D^{\star,\nu}[\hat{\psi}^\dagger]\hat{\psi}(\vec{x},t)\} - \frac{1}{\mu_0}\vec{\nabla}\times\hat{B}(\vec{x},t)|^\nu
\end{aligned}$$

The probability current is given by: $\hat{j}^\nu(\vec{x},t) = -i\frac{\hbar}{2m}\{\hat{\psi}^\dagger(\vec{x},t)D^\nu[\hat{\psi}] - D^{\star,\nu}[\hat{\psi}^\dagger]\hat{\psi}(\vec{x},t)\}$, one can recognize the Maxwell-Ampère equation:

$$\vec{\nabla}\times\hat{B}(\vec{x},t)|^\nu = \mu_0 q\hat{j}^\nu(\vec{x},t) + \frac{1}{c^2}\frac{d}{dt}\hat{E}^\nu(\vec{x},t)$$

IV. THE POWER-ZIENAU-WOOLLEY HAMILTONIAN CREATES NON-PHYSICAL STATES.

We have shown in the main text of this article that the Power-Zienau-Woolley hamiltonian density reduces to an expression involving terms written in the Poincaré gauge (the kinetic energy density term) and other terms written in the Coulomb gauge (the electromagnetic energy density term). We recall here the expression of the Power-Zienau-Woolley hamiltonian:

$$\hat{\mathcal{H}}_{pzw} = \hat{\xi}^\dagger(\vec{x},t)\{(-\frac{\hbar^2}{2m})[\vec{\nabla} - \frac{iq}{\hbar}\hat{A}_p(\vec{x},t)]^2 + V(\vec{x},t)\}\hat{\xi}(\vec{x},t) + [\frac{1}{2}\varepsilon_0\partial_t\hat{A}_c^\perp(\vec{x},t)^2 + \frac{1}{2}\varepsilon_0\vec{\nabla}\hat{\phi}_c(\vec{x},t)^2 + \frac{1}{2\mu_0}\hat{B}^2(\vec{x},t)]$$

With $\hat{A}_p(\vec{x},t) = -\vec{x}\times\int_0^1 udu\hat{B}(u\vec{x},t)$, being the vector potential in the Poincaré gauge whereas $\hat{A}_c^\perp(\vec{x},t)$ is the vector potential in the Coulomb gauge.

In the Power *et. al* derivation, the gauge is fixed to the Coulomb gauge[1, 2, 5, 7–10, 12, 17]. The vector potential in the Coulomb gauge satisfied the wave equation $\Delta\hat{A}_c = \frac{1}{c^2}\frac{\partial^2}{\partial t^2}\hat{A}_c$. The most general solution is given by a Fourier transformation since this is a natural basis for the wave equation. Annihilation/creation operators are defined with

the help of the canonical commutation relations. The expansion of the vector potential that satisfies the Coulomb gauge condition $\vec{\nabla}.\hat{A}(\vec{x},t)=0$ reads (see *e.g.* [14] p.320):

$$\hat{A}_c^{\perp}(\vec{x},t)=\sum_{\rho=1,2}\int\frac{d\vec{k}}{(2\pi)^{3/2}}\mathcal{E}_{\vec{k}}\vec{e}_{\vec{k},\rho}[e^{i(\vec{k}.\vec{x}-\omega t)}\hat{a}_{\vec{k},\rho}+e^{-i(\vec{k}.\vec{x}-\omega t)}\hat{a}^{\dagger}_{\vec{k},\rho}]$$

Where $\vec{e}_{\vec{k},\rho}$ are two orthonormal-polarization vectors ($\rho=1,2$) satisfying $\vec{k}.\vec{e}_{k,\rho}=0$. The annihilation/creation operator $\hat{a}_{\vec{k},\rho}$, $\hat{a}^{\dagger}_{\vec{k},\rho}$ respectively annihilates or creates modes with wavevector $\vec{k}$ and polarisation state $\vec{e}_{\vec{k},\rho}$.

The mode volume is $\mathcal{E}_{\vec{k}}=\sqrt{\frac{\varepsilon_0}{2\hbar\omega}}$. The angular frequency is given by $\omega=c|\vec{k}|$ where c is the speed of light in vacuum.

In order to satisfy the canonical commutation rules in the Coulomb gauge $[\hat{A}^{\perp}_{\mu}(\vec{x},t),\hat{\pi}^{\perp}_{\nu}(\vec{y},t)]=i\hbar\delta^{\perp}_{m,n}(\vec{x}-\vec{y})$, the annihilation and creation operators satisfy the following commutator:

$$[\hat{a}^{\dagger}_{\vec{k},\rho},\hat{a}^{\dagger}_{\vec{k}',\rho'}]=\delta_{\rho,\rho'}\delta(\vec{k}-\vec{k}')$$

The magnetic reads:

$$\begin{aligned}\hat{B}(\vec{x},t)&=\vec{\nabla}\times\hat{A}_c^{\perp}(\vec{x},t)\\ \hat{B}(\vec{x},t)&=i\sum_{\rho=1,2}\int\frac{d\vec{k}}{(2\pi)^{3/2}}\mathcal{E}_{\vec{k}}(\vec{k}\times\vec{e}_{\vec{k},\rho})[e^{i(\vec{k}.\vec{x}-\omega t)}\hat{a}_{\vec{k},\rho}-e^{-i(\vec{k}.\vec{x}-\omega t)}\hat{a}^{\dagger}_{\vec{k},\rho}]\end{aligned}\tag{25}$$

The Fock space generated by the action of the creation operators on the vacuum state $|0\rangle$ contains only states with transverse polarization. We call these states *the physical states*[16].

We will show now that the photonics operator $\hat{A}_p(\vec{x},t)$ generates longitudinal electromagnetic states that do not belong to the Fock space.

The photonics operator $\hat{A}_p(\vec{x},t)$ can be expressed with the help of creation/annihilation operators as:

$$\begin{aligned}\hat{A}_p(\vec{x},t)&=-\vec{x}\times\int_0^1 udu\hat{B}(u\vec{x},t)\\ &=i\sum_{\rho=1,2}\int\frac{d\vec{k}}{(2\pi)^{3/2}}\vec{x}\times(\vec{k}\times\vec{e}_{\vec{k},\rho})\int_0^1 udu\mathcal{E}_{\vec{k}}[e^{i(\vec{k}.u\vec{x}-\omega t)}\hat{a}_{\vec{k},\rho}-e^{-i(\vec{k}.u\vec{x}-\omega t)}\hat{a}^{\dagger}_{\vec{k},\rho}]\end{aligned}$$

We apply this operator to the vacuum state $|0\rangle$. Using $\vec{x}\times(\vec{k}\times\vec{e}_{\vec{k},\rho})=(\vec{x}.\vec{e}_{\vec{k},\rho})\vec{k}-(\vec{x}.\vec{k})\vec{e}_{\vec{k},\rho}$, we obtain:

$$\begin{aligned}\hat{A}_p(\vec{x},t)\left|0\right\rangle&=-i\sum_{\rho=1,2}\Big\{\int\frac{d\vec{k}}{(2\pi)^{3/2}}(\vec{x}.\vec{e}_{\vec{k},\rho})\vec{k}\int_0^1 udu\mathcal{E}_{\vec{k}}e^{-i(\vec{k}.u\vec{x}-\omega t)}\left|1;\vec{k},\rho\right\rangle-\\ &\int\frac{d\vec{k}}{(2\pi)^{3/2}}(\vec{x}.\vec{k})\vec{e}_{\vec{k},\rho}\int_0^1 udu\mathcal{E}_{\vec{k}}e^{-i(\vec{k}.u\vec{x}-\omega t)}\left|1;\vec{k},\rho\right\rangle\Big\}\end{aligned}$$

$|1;\vec{k},\rho\rangle$ is a single photon state characterized by a wavevector $\vec{k}$ and the polarization vector $\vec{e}_{\rho}$.

This expression holds for all positions $\vec{x}$. So in the general case, $\vec{x}.\vec{e}_{\vec{k},\rho}\neq 0$. The photonics operator $\hat{A}_p(\vec{x},t)$ creates electromagnetic states parallel to the wave-vector, *i.e.* longitudinal photon-states. Theses states don't belong to the Fock space. There are not physical states. So, this photonics operator creates non-physical states *i.e.* states that do not satisfy the gauge constraints. We recall that the hamiltonian under study describes one single electron interacting with the electromagnetic field. There is no longitudinal-polarization is such a situation.

These non-physical states with longitudinal polarization arises because in the Poincaré gauge, the vector potential is neither transverse nor longitudinal. As a consequence, this photonics operator creates electromagnetic states that do not belong to the Fock space generated by the annihilation/creation operators in the Coulomb gauge. This is a

consequence of the Power-Zienau-Woolley derivation that breaks the gauge-symmetry of electromagnetism and does not leave invariant the sub-manifold of physical states.

---

[1] M. Babiker and R. Loudon, Proceedings of the Royal Society of London A: Mathematical, Physical and Engineering Sciences **385**, 439 (1983), ISSN 0080-4630.
[2] C. Cohen-Tannoudji, J. Dupont-Roc, and G. Grynberg, *Atom—Photon Interactions* (Wiley-VCH Verlag GmbH, 2008).
[3] J. D. Jackson, American Journal of Physics **70**, 917 (2002).
[4] D. H. Kobe, American Journal of Physics **56**, 252 (1988).
[5] E. A. Power and S. Zienau, Philosophical Transactions of the Royal Society of London A: Mathematical, Physical and Engineering Sciences **251**, 427 (1959), ISSN 0080-4614.
[6] E. A. Power and T. Thirunamachandran, Proceedings of the Royal Society of London A: Mathematical, Physical and Engineering Sciences **372**, 265 (1980), ISSN 0080-4630.
[7] R. G. Woolley, Journal of Physics B: Atomic and Molecular Physics **7**, 488 (1974), URL `http://stacks.iop.org/0022-3700/7/i=4/a=023`.
[8] R. G. Woolley, Proceedings of the Royal Society of London A: Mathematical, Physical and Engineering Sciences **321**, 557 (1971), ISSN 0080-4630.
[9] E. A. Power and T. Thirunamachandran, Phys. Rev. A **28**, 2649 (1983), URL `http://link.aps.org/doi/10.1103/PhysRevA.28.2649`.
[10] J. R. Ackerhalt and P. W. Milonni, J. Opt. Soc. Am. B **1**, 116 (1984), URL `http://josab.osa.org/abstract.cfm?URI=josab-1-1-116`.
[11] P. W. Milonni, *The Quantum Vacuum* (Academic Press, San Diego, 1994), ISBN 978-0-08-057149-2, URL `http://www.sciencedirect.com/science/article/pii/B9780080571492500047`.
[12] G. Compagno, R. Passante, and F. Persico, *Atom-Field Interactions and Dressed Atoms* (Cambridge University Press, 1995), ISBN 9780511599774, cambridge Books Online, URL `http://dx.doi.org/10.1017/CBO9780511599774`.
[13] S. Weinberg, *The Quantum Theory of Fields*, vol. 1 (Cambridge University Press, 2005).
[14] Weinberg, *Lectures on quantum mechanics* (Cambridge University Press, 2013).
[15] H. Weyl, *Theory of Groups and Quantum Mechanics* (Dover, New York, 1950, 1931), 2nd ed.
[16] Derendinger, *Theorie quantique des champs* (Presses polytechniques et universitaires romandes, 2001).
[17] E. A. Power and T. Thirunamachandran, J. Opt. Soc. Am. B **2**, 1100 (1985), URL `http://josab.osa.org/abstract.cfm?URI=josab-2-7-1100`.
[18] P. A. M. Dirac, *The Principles of quantum Mechanics* (Oxford University Press, 1958), 3rd ed.
[19] J. Zinn-Justin and R. Guida, Scholarpedia **3**, 8287 (2008).
[20] P. A. M. Dirac, *Lectures on quantum mechanics* (Dover Publications, 2001).
